# LRRK2 Mediates α-Synuclein-Induced Neuroinflammation and Ferroptosis through the p62-Keap1-Nrf2 Pathway in Parkinson’s Disease

**DOI:** 10.1007/s10753-025-02291-8

**Published:** 2025-04-02

**Authors:** Xinjie Liu, Zijian Zheng, Cheng Xue, Xiangrong Wang, Jianwei Li, Zheng Liu, Wenqiang Xin, Xinping Xu, Dongwei Zhou, Longping Yao, Guohui Lu

**Affiliations:** 1 Department of Neurosurgery, The First Affiliated Hospital, Jiangxi Medical College, Nanchang University, Nanchang, 330006, Jiangxi China; 2 Department of Neuroanatomy, Group for Regeneration and Reprogramming, Institute for Anatomy and Cell Biology, Medical Faculty, Heidelberg University, Heidelberg, Germany; 3 Jiangxi Institute of Respiratory Disease, the First Affiliated Hospital of Nanchang University, Nanchang, Jiangxi China

**Keywords:** Parkinson’s disease, LRRK2, Ferroptosis, p62-Keap1-Nrf2 pathway, Neuroinflammation

## Abstract

Parkinson’s disease (PD) is the second most prevalent neurodegenerative disorder worldwide, characterized by the progressive degeneration of dopaminergic neurons in the substantia nigra pars compacta and the abnormal aggregation of α-synuclein (α-syn). Despite extensive research, the mechanisms underlying microglial-mediated neuroinflammation and ferroptosis in PD remain inadequately understood. In particular, the role of leucine-rich repeat kinase 2 (LRRK2) in microglial cells and its modulation of the p62-Keap1-Nrf2 signaling pathway warrant further investigation.In this study, we present novel findings demonstrating that LRRK2 regulates microglial neuroinflammation and ferroptosis through the p62-Keap1-Nrf2 signaling axis in the context of PD. Using α-syn-stimulated BV2 microglial cells, we found that LRRK2 inhibition significantly reduced the production of pro-inflammatory cytokines and enhanced the activation of the p62-Keap1-Nrf2 pathway, thereby mitigating ferroptosis and oxidative stress. Furthermore, conditioned medium from LRRK2-inhibited microglia conferred neuroprotective effects on cultured neurons, highlighting the therapeutic potential of targeting LRRK2 in microglia.Importantly, these in vitro findings were corroborated in the MPTP-induced PD mouse model, where LRRK2 inhibition led to diminished microglial activation, decreased apoptosis of midbrain dopaminergic neurons, and upregulation of the p62-Keap1-Nrf2 pathway.Our study fills a critical gap in understanding the microglial mechanisms mediated by LRRK2 and provides novel insights into the pathogenesis of PD. These findings suggest that targeting LRRK2 in microglia may represent a promising therapeutic strategy for PD.

**Supplementary Information:**

The online version contains supplementary material available at 10.1007/s10753-025-02291-8.

## Introduction

Parkinson’s Disease (PD) stands as the second most prevalent neurodegenerative disorder, typified by the progressive apoptosis of dopaminergic neurons in the substantia nigra pars compacta (SNpc) and the anomalous aggregation of α-synuclein [1, 2]. Despite the prominence of these pathological hallmarks, the precise interrelationship between them remains elusive. The underlying molecular mechanisms of PD pathogenesis encompass a myriad of pathways, including α-synuclein homeostasis, mitochondrial function, oxidative stress, calcium homeostasis, axonal transport, and neuroinflammation [3]. In recent years, mounting evidence has underscored the pivotal role of chronic central nervous system (CNS) inflammation in the progression of PD pathology [4–6]. Microglia, the resident immune cells of the brain, are widely recognized as pivotal players in the CNS immune response, acting akin to macrophages. As key modulators of neuroinflammation through their phenotypic transformation, microglia are increasingly implicated in PD pathogenesis [7]. Ample evidence correlates glial activation with PD, yet the molecular mechanisms governing these interactions are not fully delineated. Molecular and clinical findings, including positron emission tomography (PET) imaging, have revealed pronounced microglial activation in the substantia nigra of PD patients [8, 9]. Once activated, microglia can trigger the activation of NADPH oxidase and NF-κB pathways, secretion of pro-inflammatory mediators, and elevation of reactive oxygen species (ROS), ultimately precipitating dopaminergic neuron apoptosis [10, 11]. Unraveling the links between microglial activation and neuroinflammation is crucial for elucidating the mechanisms of dopaminergic neuron death, potentially offering new therapeutic targets to mitigate PD progression.

Leucine-rich repeat kinase 2 (LRRK2) is recognized as one of the most prevalent genetic risk factors for PD [12]. LRRK2 is a large, multidomain protein, with its N-terminal comprising armadillo, ankyrin, and leucine-rich repeat domains, while its C-terminal contains two catalytic domains: a GTPase domain and a serine/threonine kinase domain. Additionally, it includes two scaffold domains: a C-terminal of ROC (COR) domain positioned between the GTPase and kinase domains, and a terminal WD40 domain [13]. Enhanced LRRK2 kinase activity is a feature observed in both familial and sporadic PD cases. Clinically, PD patients harboring LRRK2 mutations are often phenotypically indistinguishable from those with idiopathic PD, indicating common pathological mechanisms. Beyond PD, LRRK2 is implicated in various inflammatory diseases such as inflammatory bowel disease, tuberculosis, and leprosy, underscoring its crucial role in inflammation [14, 15]. LRRK2 is highly expressed in peripheral immune cells, where its expression is tightly regulated by immune stimuli. The absence of LRRK2 suppresses immune responses. Notably, within the CNS, the deletion of LRRK2 in microglia increases the uptake and clearance of α-synuclein [16, 17]. Inhibiting LRRK2 or its kinase activity in primary microglial cultures reduces the production of pro-inflammatory cytokines including tumor necrosis factor (TNF) and interleukin-1β (IL-1β) [18, 19]. Moreover, LRRK2 expression and kinase activity are upregulated in microglia upon lipopolysaccharide (LPS) injection in CNS models,[20]suggesting that LRRK2 potentially facilitates neuroinflammation and PD pathology. Despite these insights, the precise mechanisms by which elevated LRRK2 kinase activity leads to microglial activation and subsequent neuroinflammation remain unclear. Deciphering LRRK2’s role in PD pathogenesis is thus of paramount importance, as it may reveal the pathological mechanisms of LRRK2-related PD and provide critical insights for the development of therapeutic strategies aimed at mitigating PD progression.

Ferroptosis, a distinct form of regulated cell death, differs fundamentally from conventional pathways such as apoptosis or necroptosis [21]. It is characterized by its iron dependence and lethal accumulation of lipid peroxides. The hallmark features of ferroptosis include increased lipid peroxides and decreased activity of glutathione peroxidase 4 (GPX4) [22]. An increasing body of research indicates that ferroptosis plays a significant role in neurodegeneration, particularly emphasizing that elevated iron levels and metabolic dysregulation in the substantia nigra are crucial in PD [23–25]. Furthermore, elevated lipid peroxidation has not only been observed in the substantia nigra pars compacta of PD patients but also in their cerebrospinal fluid and plasma [26–28]. These findings suggest a strong correlation between ferroptosis and the death of dopaminergic neurons in PD.

Nuclear factor erythroid 2-related factor 2 (Nrf2) is a transcription factor belonging to the basic leucine zipper (bZIP) protein family, which plays a crucial role in protecting cells from oxidative damage by activating the expression of detoxifying enzymes and antioxidant proteins [29]. Kelch-like ECH-associated protein 1 (Keap1) is a cysteine-rich protein consisting of 624 amino acids and contains multiple cysteine residues that act as sensors for various stress stimuli [30]. Upon exposure to electrophiles, ROS, or chemical stressors, the cysteine residues of Keap1 undergo chemical modifications, inducing conformational changes that result in the dissociation and activation of Nrf2. The activated Nrf2 then translocates into the nucleus, where it initiates the transcription and expression of downstream target genes including phase II detoxifying enzymes, antioxidant proteins, and proteasome/chaperone proteins to counteract harmful internal and external stimuli [31]. p62/SQSTM1 (hereafter referred to as p62) is a stress-induced intracellular protein widely recognized as a receptor for ubiquitinated proteins, microbes, and organelles [32]. When p62 accumulates intracellularly, it competes with Nrf2 for binding to Keap1, leading to the dissociation of Nrf2 from Keap1 and subsequent nuclear translocation, thereby initiating the expression of downstream protective genes. Previous studies have shown that the p62-Keap1-Nrf2 pathway plays a critical role in the regulation of ferroptosis, tumor resistance, and oxidative stress. In this study, we innovatively explore the hypothesis that LRRK2 regulates microglial neuroinflammation and ferroptosis through the p62-Keap1-Nrf2 pathway in PD. By elucidating this mechanism, we aim to address a significant gap in the current understanding of PD pathogenesis and to provide a foundation for developing novel therapeutic interventions targeting microglial LRRK2.

## Methods

### Cell Culture and Treatment

In this study, we utilized two cellular models to investigate neuroinflammatory processes and dopaminergic cell death: the murine microglial cell line BV2 for neuroinflammation studies, and the human neuroblastoma cell line SH-SY5Y for examining dopaminergic neuronal apoptosis. Both cell lines were obtained from the Central Laboratory of the First Affiliated Hospital of Nanchang University (Nanchang, China). BV2 cells were maintained in Dulbecco’s Modified Eagle Medium (DMEM; 11,965,084; Gibco), while SH-SY5Y cells were cultured in DMEM/F12 medium (11,330,032, Gibco). Both media were supplemented with 10% heat-inactivated fetal bovine serum (FBS; 10,099,141; Gibco) and 1% penicillin–streptomycin (100 U/mL penicillin and 100 μg/mL streptomycin; 15,140,122; Gibco). Cells were maintained at 37 °C in a humidified atmosphere containing 5% CO_2_ and passaged every 2–3 days when reaching 80–90% confluence. For experimental treatments, BV2 cells were seeded at a density of 1 × 10^5^ cells/mL and allowed to adhere for 24 h prior to treatment. Neuroinflammation was induced by exposing cells to recombinant human α-synuclein protein (10 μg/mL; S7820; Sigma-Aldrich). LRRK2 kinase activity was inhibited using PF-06447475 (2 μg/mL; PZ0185; Sigma-Aldrich). To investigate ferroptotic mechanisms, cells were treated with either the ferroptosis inducer Erastin (10 μM; E7781, Sigma-Aldrich) or the ferroptosis inhibitor Ferrostatin-1 (0.5 μM; SML0583; Sigma-Aldrich). All pharmacological agents were reconstituted and stored according to the manufacturer’s specifications.

### Experimental Animals and Treatments

Male C57BL/6 mice (8 weeks old, 22–25 g) were purchased from the Laboratory Animal Center of Nanchang University and maintained under controlled environmental conditions (22 ± 2 °C temperature, 55 ± 5% humidity, and a 12-h light/dark cycle) with ad libitum access to standard chow and water. Following a one-week acclimatization period, a total of 84 mice were included in the final analyses. All procedures involving animals were reviewed and approved by the Nanchang University Ethics Committee [Ethics Approval No. (2023)CDYFYYLK(05–017)].

To establish the Parkinson’s disease model, mice received intraperitoneal injections of MPTP-HCl (30 mg/kg body weight; Sigma-Aldrich) once daily for five consecutive days, whereas control animals received equivalent volumes of sterile saline (0.9% NaCl). PF-06447475 (20 mg/kg, twice daily) was administered via oral gavage, beginning two days prior to MPTP treatment and continuing throughout the 14-day experimental period. Oral gavage was performed using a 20 G blunt-ended stainless steel feeding needle (approximately 5 cm in length; sterilized before each use), ensuring accurate and safe drug delivery. This dosing protocol was previously optimized based on pilot studies to achieve maximal LRRK2 inhibition [33].

The study comprised two separate experiments. In Experiment 1, mice were randomly allocated to four groups: Saline control, 0 days post-MPTP, 7 days post-MPTP, and 21 days post-MPTP. In Experiment 2, mice were allocated to three groups: Saline control, MPTP, and MPTP + PF-06447475. Each group contained an equal number of animals, resulting in a total of 84 mice across both experiments. The cohort was systematically divided to facilitate multiple analytical procedures, including behavioral assessments and immunohistochemical evaluations (42 mice), Western blot analyses (21 mice), and RNA extraction followed by reverse-transcription quantitative real-time PCR (RT-qPCR) (21 mice) (Figures S1, Supporting Information).

At the conclusion of the experimental period, all mice were euthanized, and the substantia nigra pars compacta (SNpc) was rapidly dissected. Tissues were either processed immediately or flash-frozen in liquid nitrogen and stored at − 80 °C for subsequent analyses. For behavioral and immunohistochemical assays, 6 mice per group were used. For molecular and biochemical analyses, tissues were obtained from three independent animals per group, with all measurements performed in technical triplicates (see Supplementary Table S1).

### Primary Microglia Extraction and Culture

Murine primary microglial cells were isolated from 1–3-day-old C57BL/6 mice (Laboratory Animal Center of Nanchang University, China) in accordance with the guidelines approved by the Nanchang University Ethics Committee. Briefly, whole brains were carefully dissected under aseptic conditions and transferred to ice-cold phosphate-buffered saline (PBS; pH 7.4). Meninges and visible blood vessels were meticulously removed, and the remaining cortical tissue was mechanically minced into small fragments before enzymatic digestion with 0.25% trypsin (Servicebio; G4012) at 37 °C for 15 min to liberate individual cells. The digestion was halted by adding an equal volume of Dulbecco’s Modified Eagle Medium (DMEM; 11,965,084, Gibco) supplemented with 10% heat-inactivated fetal bovine serum (FBS; 10,099,141, Gibco) and 1% penicillin–streptomycin (15,140,122, Gibco). The cell suspension was passed through a 70-μm cell strainer to remove debris, centrifuged at 200 × g for 5 min, and resuspended in fresh DMEM containing 10% FBS and 1% penicillin–streptomycin. Subsequently, the cells were plated into T75 flasks and maintained at 37 °C in a humidified 5% CO_2_ incubator, with medium changes every 3 days. After approximately 10–12 days, when the mixed glial cultures reached confluence, the flasks were gently shaken at 200 rpm for 4 h at 37 °C to dislodge the loosely adherent microglia, which were collected from the supernatant. The harvested cells were centrifuged (200 × g, 5 min), resuspended in fresh DMEM supplemented with 10% FBS and 1% penicillin–streptomycin, and reseeded in appropriate culture plates or dishes for subsequent experiments. The isolated microglial population was assessed using immunostaining with the microglial marker Iba1, demonstrating that microglia consistently accounted for more than 95% of the cultured cells (Figures S2, Supporting Information).

### RNA Extraction and Quantitative RT–PCR Analysis

Total RNA was isolated from BV2 cells utilizing the EZ-PRESS RNA Purification Kit (EZ-Bioscience) in accordance with the manufacturer’s instructions. The mRNA expression levels of TNF-α, IL-1β, IL-6, LRRK2, p62, Keap1, and Nrf2 were subsequently determined. The quantity and quality of extracted RNA were assessed using a NanoDrop™ spectrophotometer (Thermo Fisher Scientific), with samples exhibiting A260/A280 ratios between 1.8 and 2.0 being selected for subsequent analysis. Complementary DNA (cDNA) was synthesized from 1 μg of total RNA using the PrimeScript™ RT Reagent Kit (Takara Bio) under the following conditions: 37 °C for 15 min followed by 85 °C for 5 s. RT-qPCR was performed using SYBR® Premix Ex Taq™ II (Takara Bio) on a CFX96 Real-Time PCR Detection System (Bio-Rad Laboratories). The relative expression levels of target genes were normalized to glyceraldehyde 3-phosphate dehydrogenase (GAPDH) as an internal reference gene. The ΔΔCt method was utilized to compute and normalize the relative expression of each gene.

### Western Blot

Western blotting was employed to determine the expression levels of proteins LRRK2, p62, Keap1, Nrf2, GPX4, p-p65, p65, p-rab10 and β-actin, in BV2 cells and mice. Cells were harvested and lysed in RIPA buffer (P0013B; Beyotime) supplemented with protease and phosphatase inhibitors (B14001 and B15001; Biotools). Protein concentrations were quantified using the Bicinchoninic Acid (BCA) Protein Assay Kit (Thermo Fisher Scientific). Equal amounts of protein (20 μg) from each sample were separated by electrophoresis on 10% SDS-PAGE gels and transferred to polyvinylidene difluoride (PVDF) membranes (Solarbio). The membranes were blocked in 5% non-fat dry milk dissolved in Tris-buffered saline containing 0.1% Tween-20 (TBST) for 1 h at room temperature and then incubated overnight at 4 °C with specific primary antibodies: anti-LRRK2 (ab133474; Abcam; 1:10,000), anti-p62 (D6M5X; Cell Signaling Technology; 1:1000), anti-Keap1 (ab227828; Abcam; 1:2000), anti-Nrf2 (D1Z9C; Cell Signaling Technology; 1:1000), anti-GPX4 (ab125066; Abcam; 1:1000), anti-p-p65 (Ser536; Cell Signaling Technology; 1:1000), anti-p65 (D14E12; Cell Signaling Technology; 1:1000), anti-p-rab10 (phospho T73; ab230261; Abcam; 1:1000) and anti-β-actin (ab8227; Abcam; 1:1000) as an internal loading control. Following primary antibody incubation, membranes were washed with TBST and incubated with horseradish peroxidase (HRP)-conjugated secondary antibodies (1:5000; Cell Signaling Technology) for 1 h at room temperature. The immunoreactive bands were detected using an enhanced chemiluminescence (ECL) kit (Thermo Fisher Scientific) and densitometric analysis of band intensities was performed using ImageJ software, and the protein expression levels were normalized to those of β-actin.

### Cell Viability Assessment

Cell viability was determined using the Cell Counting Kit-8 (CCK-8) assay (96,992; Sigma-Aldrich) according to the manufacturer’s instructions. BV2 microglial cells were seeded into 96-well plates at a density of 5 × 10^3^ cells per well and allowed to adhere overnight. Following treatment with α-synuclein (10 μg/mL), PF-06447475 (2 μg/mL), Erastin (10 μM), or Ferrostatin-1 (0.5 μM) for 24 h, 10 μL of CCK-8 solution was added to each well containing 100 μL of culture medium. After 2 h incubation at 37 °C in a humidified atmosphere containing 5% CO_2_, the absorbance was measured at 450 nm using a microplate reader (SpectraMax M5, Molecular Devices). Cell viability was expressed as a percentage relative to the control group. Each experiment was performed in triplicate.

### Transfection

To overexpress LRRK2 and p62 in BV2 microglial cells, we utilized plasmid constructs encoding the mouse LRRK2 (NM_025730) and p62 (NM_011018) genes, which were obtained from OriGene Technologies (Rockville). BV2 cells were seeded in 6-well plates at a density of 1 × 10^5^ cells per well and allowed to adhere overnight under standard culture conditions (37 °C, 5% CO2) in Dulbecco’s Modified Eagle Medium (DMEM) supplemented with 10% fetal bovine serum and 1% penicillin–streptomycin. On the day of transfection, the culture medium was replaced with fresh DMEM, and the cells were transfected with 4 μg of the respective plasmid DNA (LRRK2 or p62) using Lipofectamine 2000 transfection reagent (Thermo Fisher Scientific) according to the manufacturer’s instructions. After 6 h of incubation, the transfection medium was replaced with complete growth medium, and the cells were cultured for an additional 48 h before being harvested for further analyses. The efficiency of LRRK2 and p62 overexpression was confirmed by Western blot analysis.

### CD40 Expression Analysis

BV2 microglial cells were seeded in 12-well plates at a density of 2 × 10^5^ cells per well and cultured until reaching 70–80% confluency in complete growth medium under standard conditions (37 °C, 5% CO2). Cells were then subjected to various treatments including α-synuclein (10 μg/mL), PF-06447475 (2 μg/mL), Erastin (10 μM), and Ferrostatin-1 (0.5 μM) according to the experimental design parameters. Following the treatment period, cells were harvested using non-enzymatic cell dissociation solution to preserve surface protein integrity, and subsequently washed with ice-cold flow cytometry staining buffer (phosphate-buffered saline containing 2% fetal bovine serum). Surface staining was performed by incubating cells with FITC-conjugated anti-mouse CD40 monoclonal antibody (124,608; BioLegend) for 15 min at 4 °C in the dark. After incubation, cells were washed twice with staining buffer to remove unbound antibodies and resuspended in 500 μL of flow cytometry buffer supplemented. Cell fluorescence analysis was performed using an LSRII flow cytometer (BD Biosciences). Data acquisition and analysis were conducted using FlowJo software (TreeStar).

### Enzyme-Linked Immunosorbent Assay (ELISA)

The concentrations of pro-inflammatory cytokines in cell culture supernatants were quantified using commercial ELISA kits. Briefly, BV2 microglial cells were seeded in 24-well plates at a density of 1 × 10^5^ cells per well and allowed to adhere overnight. Following treatment with α-synuclein, PF-06447475, Erastin, or Ferrostatin-1 for 24 h, culture supernatants were collected and centrifuged at 1,000 × g for 10 min at 4 °C to remove cellular debris. The levels of pro-inflammatory cytokines, including IL-6 (PI326; Beyotime), tumor necrosis factor-alpha (TNF‐⍺) (PT512; Beyotime), and IL-1β (PI301; Beyotime), were measured according to the manufacturer’s protocols. The absorbance was measured at 450 nm with wavelength correction at 570 nm using a microplate reader (SpectraMax M5, Molecular Devices). All samples were analyzed in triplicate, and results were expressed as pg/mL.

### Quantification of Intracellular Iron

Mouse BV2 microglial cells were cultured in 12-well plates at a density of 2 × 10^5^ cells per well and allowed to reach 70–80% confluency. Cells were then subjected to treatment with α-synuclein (10 μg/mL), PF-06447475 (2 μg/mL), Erastin (10 μM), and Ferrostatin-1 (0.5 μM) according to the experimental protocol. Following treatment, cells were lysed, and cell lysates were collected by centrifugation. Intracellular iron levels were quantified using an Iron Assay Kit (Sigma‐Aldrich; MAK025) following the manufacturer’s instructions. Absorbance was measured at 595 nm using a microplate reader.

### Measurement of ROS Production

Mouse BV2 microglial cells were seeded in 12-well plates at a density of 2 × 10^5^ cells per well and cultured until 70–80% confluency was reached. Cells were then treated with α-synuclein (10 μg/mL), PF-06447475 (2 μg/mL), Erastin (10 μM), and Ferrostatin-1 (0.5 μM) based on experimental design. ROS production was assessed using DCFDA-DA (Sigma‐Aldrich). Cells were incubated with 10 μM DCFDA for 30 min at 37 °C. Fluorescence intensity was then measured using an LSRII cytometer (BD Biosciences). Data were analyzed using the FlowJo software program.

### Measurement of Malondialdehyde (MDA) Levels

Mouse BV2 microglial cells were seeded in 12-well plates at a density of 2 × 10^5^ cells per well and cultured until reaching 70–80% confluency. Cells were then treated with α-synuclein (10 μg/mL), PF-06447475 (2 μg/mL), Erastin (10 μM), and Ferrostatin-1 (0.5 μM) as per experimental requirements. Post-treatment, cells were harvested, and MDA levels were measured to assess lipid peroxidation. Cell lysates were prepared, and MDA levels were determined using a Lipid Peroxidation (MDA) Assay Kit (Sigma-Aldrich) following the manufacturer’s instructions. The assay involves the reaction of MDA with thiobarbituric acid (TBA) to form a colored complex, which was measured at 532 nm using a BioTek microplate reader.

### Determination of Glutathione (GSH) Levels

Mouse BV2 microglial cells were cultured in 12-well plates at a density of 2 × 10^5^ cells per well and allowed to reach 70–80% confluency. Cells were then treated with α-synuclein (10 μg/mL), PF-06447475 (2 μg/mL), Erastin (10 μM), and Ferrostatin-1 (0.5 μM) as needed for the specific experimental setup. Following treatment, cells were lysed and deproteinated, and the supernatants were analyzed using a Glutathione Assay Kit (S0053; Beyotime) according to the manufacturer’s instructions. Absorbance at 405 nm was measured using a microplate reader, and GSH concentrations were extrapolated from a standard curve and normalized to the protein concentration of the samples.

### Immunofluorescence Staining

In the in vitro experiments, the cells used for immunofluorescence staining were primary microglia. Primary microglial cells were cultured on poly-D-lysine-coated glass coverslips in 12-well plates until reaching 70–80% confluence. Following experimental treatments, cells were fixed with 4% paraformaldehyde for 30 min at room temperature and washed thoroughly with phosphate-buffered saline (PBS, pH 7.4). Cell membranes were permeabilized using PBS containing 0.03% Triton X-100, and non-specific binding was blocked with 5% goat serum albumin (C0265; Beyotime). The cells were then incubated with primary antibodies diluted in blocking solution overnight at 4 °C. After extensive washing with PBS, cells were incubated with fluorophore-conjugated secondary antibodies for 1 h at room temperature in darkness. Nuclei were counterstained with DAPI (S2110; Solarbio). Immunofluorescence images were acquired using a Leica confocal laser scanning microscope.For in vivo studies, mice were deeply anesthetized and perfused transcardially with PBS followed by ice-cold 4% paraformaldehyde. Brains were carefully dissected and post-fixed in 4% paraformaldehyde for 48 h at 4 °C, followed by cryoprotection in 30% sucrose solution for an additional 48 h at 4 °C. Mesencephalic tissues were sectioned coronally (12 μm thickness) using a Leica freezing microtome. Sections were mounted on poly-D-lysine-coated slides and processed for immunofluorescence staining. The sections were then subjected to incubation with the specified primary antibodies. Subsequently, the slices were subjected to incubation with secondary antibodies.

The following primary antibodies were used: anti-LRRK2 (1:100), anti-p62 (1:400), anti-p-p65 (1:1000), anti-Iba1 (1:500), Cy3-conjugated goat anti-rabbit IgG (GB21303; 1:200; Servicebio) and Alexa Fluor 488-conjugated goat anti-mouse IgG (GB25301; 1:200; Servicebio). Immunofluorescence was visualized using a Zeiss LSM 880 laser scanning confocal microscope. Image acquisition and analysis were performed using ZEN light software (Zeiss). Quantitative analysis of immunofluorescence intensity was conducted using ImageJ software.

### SNpc Sample Collection and Homogenization

At the conclusion of the experimental period, each mouse was euthanized, and the SNpc was carefully dissected from each animal individually. For Western blot, tissue from the SNpc of each mouse was immediately homogenized in ice-cold RIPA buffer supplemented with protease and phosphatase inhibitors. We used either a motorized homogenizer, ensuring thorough dissociation of the tissue. The homogenate was then centrifuged to collect the supernatant, and protein concentration was determined using the BCA assay.For RT-qPCR, the fresh SNpc was homogenized directly in the provided lysis buffer from the EZ-PRESS RNA Purification Kit (EZ-Bioscience). This approach allowed efficient tissue disruption and preserved RNA integrity for subsequent extraction and cDNA synthesis.

### Immunohistochemistry

Immunohistochemistry staining was performed to analyze dopaminergic neurons in the substantia nigra. Briefly, mouse brain tissues were fixed in 4% paraformaldehyde for 24 h, dehydrated through a graded ethanol series, and embedded in paraffin. Coronal brain Sects. (5 μm thickness) encompassing the SNpc region were prepared using a rotary microtome. The sections were deparaffinized with xylene, rehydrated through decreasing concentrations of ethanol, and subjected to heat-mediated antigen retrieval in citrate buffer (10 mM, pH 6.0) for 20 min. Endogenous peroxidase activity was quenched with 3% H2O2 for 10 min, followed by blocking with 5% bovine serum albumin (BSA) in PBS for 1 h at room temperature. Dopaminergic neurons were identified by overnight incubation at 4 °C with anti-tyrosine hydroxylase (TH) primary antibody (25,859–1-AP; 1:5000; Proteintech). After washing with PBS, sections were incubated with an HRP-conjugated goat anti-rabbit IgG secondary antibody (SA00001-2; Proteintech) for 1 h at room temperature. Streptavidin-HRP ABC solution (PK-4001; Vector Laboratories) was then applied according to the manufacturer’s instructions. The immunoreactive signals were visualized using 3,3′-diaminobenzidine (DAB) as a chromogen. Images were captured using a Leica confocal microscope and analyzed with ImageJ software.

### Molecular Docking

Molecular docking analysis was performed using the HDOCK server (http://h-dockphys.hust.edu.cn/) to evaluate the potential interaction between LRRK2 and p62. The three-dimensional structure of LRRK2 was retrieved from the Protein Data Bank (PDB ID: 7li4), while the p62 structure was obtained from the AlphaFold protein database. Prior to docking simulation, protein structures were processed using PyMOL software (version 2.3.0, https://pymol.org) to remove original ligands, water molecules, and other organic compounds. The structures were further optimized using the "prepare" module in Discovery Studio software for hydrogenation and protonation states adjustment. The protein–protein interactions were analyzed using LigPlus software to evaluate the two-dimensional binding forces, while the protein–protein interaction interface was examined using the "analysis interface" module in Discovery Studio. The visualization of interacting amino acid residues between LRRK2 and p62 was accomplished using PyMOL software (version 2.3.0).

### Microglial Supernatant Transfer Model

BV2 microglial cells were seeded in 6-well plates at a density of 2 × 10^5^ cells/well and cultured in DMEM supplemented with 10% FBS until reaching 70–80% confluence. Cells were then exposed to various treatments: α-synuclein (10 μg/mL), PF-06447475 (2 μg/mL), Erastin (10 μM), or Ferrostatin-1 (1 μM) for 24 h under standard culture conditions (37 °C, 5% CO_2_). Following the treatment period, conditioned media were collected and centrifuged at 1000 × g for 10 min at 4 °C to remove cellular debris. The supernatants were then filtered through 0.22 μm sterile filters.SH-SY5Y neuroblastoma cells were pre-seeded in 6-well plates at a density of 1.5 × 10^5^ cells/well and allowed to adhere for 24 h. The filtered BV2-conditioned media were mixed with fresh complete medium at a 1:1 ratio and applied to SH-SY5Y cells. Following 24 h of exposure to the conditioned media, SH-SY5Y cells were harvested and analyzed for apoptosis using Annexin V-FITC/PI double staining followed by flow cytometric analysis.

### Apoptosis Analysis

After 24 h of treatment with BV2 microglial supernatants, SH-SY5Y cells were collected for apoptosis analysis using the Annexin V-FITC/PI Apoptosis Detection Kit (LiankeBio). SH-SY5Y cells were stained with Annexin V-FITC and Propidium Iodide (PI) according to the manufacturer’s protocol provided by LiankeBio.Stained cells were then analyzed using a flow cytometer (BD Biosciences) to determine the percentage of apoptotic cells based on Annexin V-FITC and PI staining patterns.

### Mouse Behavioral Scoring System

A comprehensive scoring system was employed to assess the severity of motor and non-motor symptoms in the mouse model. The scoring criteria were as follows:

- 1 point: Mice exhibited reduced escape behavior, piloerection, yellowing and soiling of fur, hunched posture, and decreased voluntary activity.
- 2 points: In addition to the symptoms described for 1 point, mice displayed a marked reduction in voluntary activity, lethargy, and potential tremors or unstable gait.
- 4 points: Mice presented with symptoms as described for 2 points, along with an unstable gait, inability to walk in a straight line, or rotational walking.
- 6 points: Mice demonstrated lateral recumbency, paralysis of one side’s forelimb and/or hindlimb, difficulty in walking and feeding.
- 8 points: Mice exhibited complete paralysis of one side’s forelimb and/or hindlimb, spasticity of limbs, significant weight loss, and inability to feed independently.
- 10 points: Mice were near death or deceased. Scores ranging from 2 to 6 were considered indicative of successful modeling of the disease phenotype.

### Mouse Behavioral Assessments

Rotarod Test: To evaluate motor coordination and balance, mice were subjected to the rotarod test. Prior to testing, mice underwent a training session on the apparatus, maintaining a constant speed of 20 rotations per minute (rpm) for a duration of 5 min. During the test, mice were placed on an accelerating rod, with the speed gradually increasing from 4 to 40 rpm over a period of 300 s. The latency to fall, defined as the time the mice were able to remain on the accelerating rod, was recorded for each animal. Pole Test: The pole test was employed to assess motor function and coordination. A sturdy wooden pole, measuring 50 cm in length and 1 cm in diameter, was vertically affixed to a base. At the top of the pole, a wooden ball with a diameter of 2 cm was attached. Mice were carefully placed on the ball and allowed to descend the pole. The time required for the mouse’s front paws to reach the base of the pole from the moment of release was measured. Each mouse underwent three consecutive trials, with a 30-min interval between each trial. The average time across the three trials was calculated and recorded for data analysis.

### Statistical Analysis

All experiments were performed in triplicate unless otherwise specified. Data are presented as the mean ± standard deviation (SD). Statistical analyses were conducted using GraphPad Prism 9.0 software. The normality of the data distribution was assessed using the Shapiro–Wilk test. For comparisons between two groups, an unpaired two-tailed Student’s t-test was employed. For comparisons among multiple groups, one-way analysis of variance (ANOVA) followed by Tukey’s post hoc test was utilized. A p-value less than 0.05 was considered statistically significant.

## Results

### α-Synuclein Stimulation Enhances LRRK2 Expression in Microglial Cells

**Fig. 1:**
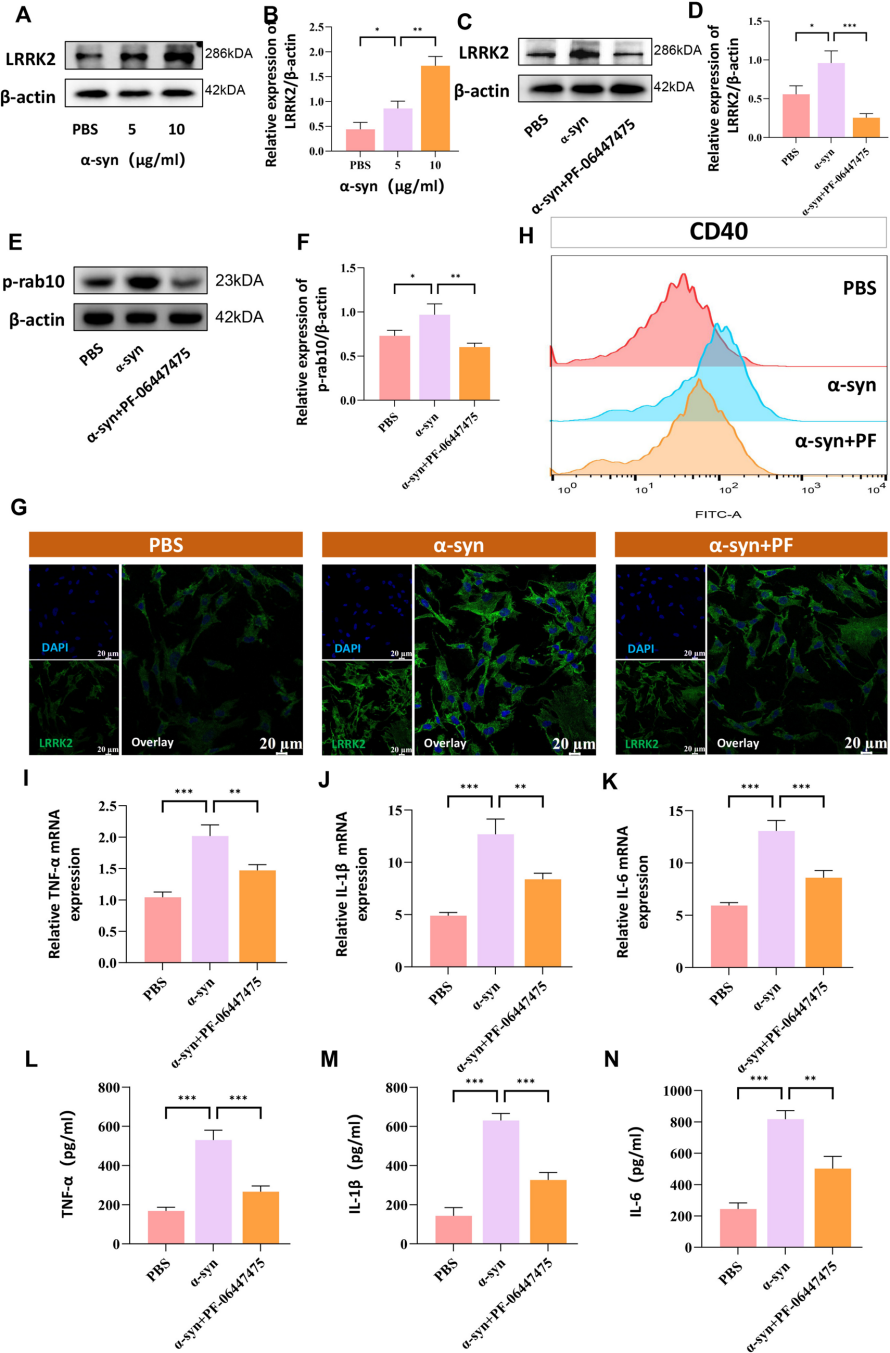
α-synuclein stimulation enhances LRRK2 expression in BV2 microglial cells. To investigate the role of LRRK2 in α-synuclein-induced microglial activation, BV2 cells were treated with α-synuclein (α-syn;5 and 10 μg/mL) for 24 h. LRRK2 protein expression was analyzed by Western blot (**A**) and quantified relative to β-actin (**B**). To evaluate the effects of LRRK2 inhibition, cells were treated with α-syn (10 μg/mL) alone or in combination with PF-06447475 (2 μg/mL) for 24 h. The expression of LRRK2 (**C**, **D**) and phosphorylated rab10 (**E**, **F**) was then analyzed by Western blotting. The expression of LRRK2 in primary microglia was further examined by immunofluorescence staining, with nuclei counterstained using DAPI (**G**). Microglial activation was assessed via flow cytometric analysis of the surface expression of the microglial activation marker CD40 (**H**). Pro-inflammatory cytokine production was measured at both mRNA level by RT-qPCR (I-K) and protein level by ELISA (**L**–**N**) for TNF-α, IL-1β, and IL-6.Data were presented as means ± SD. The experiments were carried out three times (*n* = 3). One-way analysis of variance (ANOVA) followed by Tukey’s multiple comparison tests in (**B**, **D**, **F**, **I**, **J**, **K**, **L**, **M**, **N**). The difference in folds is statistically significant. *P < 0.05, **P < 0.01, ***P < 0.001. PBS, phosphate‐buffered saline; mRNA, messenger RNA

We initially investigated the regulatory effects of α-synuclein (α-syn) on LRRK2 gene expression in BV2 microglial cells. Quantitative RT-PCR analysis revealed that LRRK2 mRNA expression exhibited both time- and dose-dependent increases following α-syn stimulation, with maximal expression observed at 24 h post-treatment (Figures S3A and 32B, Supporting Information). Specifically, exposure to α-syn at concentrations of 5 μg/ml and 10 μg/ml for 24 h resulted in significant upregulation of LRRK2 expression (Fig. 1A,B), establishing a direct correlation between α-syn exposure and LRRK2 expression in microglial cells.

### LRRK2 Inhibition Attenuates Microglial Activation and Inflammatory Response

Given the observed upregulation of LRRK2 in α-syn-stimulated microglia and previous evidence linking LRRK2 suppression to α-syn-induced neuroinflammation, we hypothesized that LRRK2 might serve as a key mediator in microglial-driven neuroinflammatory processes. To validate this hypothesis, we employed the selective LRRK2 kinase inhibitor PF-06447475. We confirmed its efficacy through multiple approaches, including Western blot analyses (Fig. 1C, D), assessment of Rab10 phosphorylation—a known LRRK2 substrate (Fig. 1E, F), and immunofluorescence staining in primary microglial cells (Fig. 1G). All these methods demonstrated a significant reduction in LRRK2 expression levels. Notably, flow cytometric analysis revealed that α-syn stimulation markedly increased the expression of CD40, a canonical microglial activation marker. This elevation was significantly attenuated following LRRK2 inhibition by PF-06447475 (Fig. 1H). Morphological assessment revealed that α-syn treatment induced characteristic features of microglial activation, including cellular hypertrophy, process retraction, and adoption of amoeboid morphology (Figure S3C). Furthermore, comprehensive analysis of inflammatory mediators demonstrated that LRRK2 inhibition significantly reduced the expression and secretion of pro-inflammatory cytokines, including TNF-α (Fig. 1I, L), IL-1β (Fig. 1J, M), and IL-6 (Fig. 1K, N), as assessed by RT-qPCR and ELISA. These findings collectively indicate that LRRK2 kinase activity is crucial for α-syn-induced microglial activation and subsequent inflammatory responses, suggesting its potential as a therapeutic target in neuroinflammatory conditions.

### α-Synuclein Induces Ferroptotic Cell Death in Microglial Cells

**Fig. 2:**
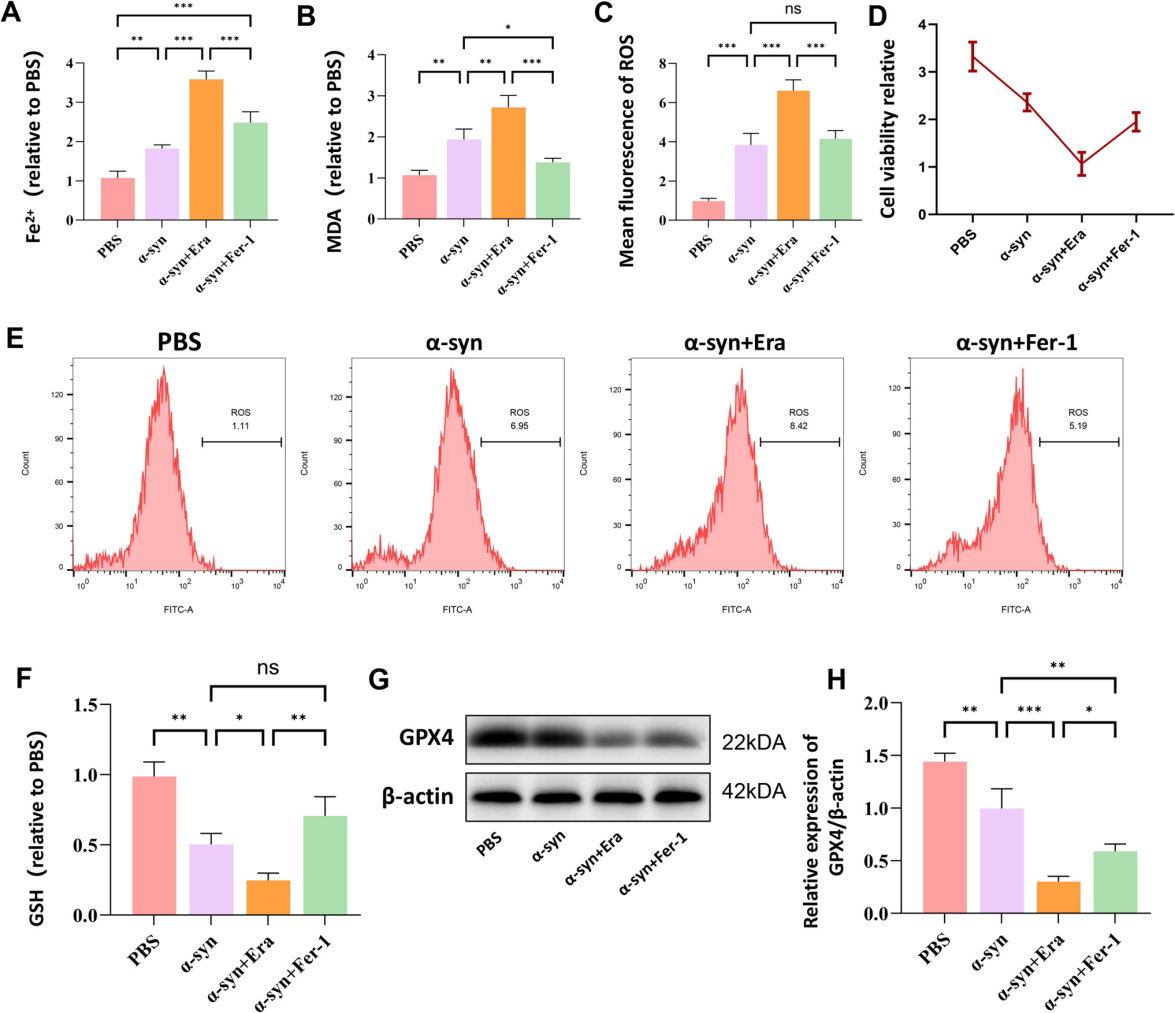
Ferroptosis mediates α-Synuclein-induced microglial activation. To examine the involvement of ferroptosis in α-syn-induced microglial activation, BV2 cells were treated with α-synuclein(α-syn;10 μg/mL), erastin (Era;10 μM), or ferrostatin-1 (Fer-1; 1 μM) for 24 h. Ferroptotic parameters were assessed including intracellular Fe^2+^ levels using Iron Assay Kit (**A**), lipid peroxidation by measuring MDA content (**B**), cellular ROS levels by DCFH-DA staining and flow cytometry with quantification (**C**, **E**), cell viability by CCK-8 assay (**D**), and intracellular GSH levels using GSH/GSSG Ratio Detection Kit (**F**). GPX4 protein expression was analyzed by Western blot and quantified relative to β-actin (**G**, **H**).Data were presented as means ± SD. The experiments were carried out three times (*n* = 3). One-way analysis of variance (ANOVA) followed by Tukey’s multiple comparison tests in (**A**, **B**, **C**, **F**, **H**). The difference in folds is statistically significant. *P < 0.05, **P < 0.01, ***P < 0.001. PBS, phosphate‐buffered saline; MDA, Malondialdehyde; GSH, glutathione

Recent evidence has implicated ferroptosis, an iron-dependent form of regulated cell death characterized by excessive lipid peroxidation, in the pathogenesis of Parkinson’s disease. To elucidate the potential role of ferroptosis in α-syn-induced microglial activation, we conducted a comprehensive analysis of ferroptotic parameters following treatment with erastin (a ferroptosis inducer) or ferrostatin-1 (a specific ferroptosis inhibitor). Our results demonstrated that erastin treatment significantly potentiated α-syn-induced microglial activation, as evidenced by multiple ferroptotic indicators: elevated intracellular Fe^2+^ levels (Fig. 2A), increased MDA content (Fig. 2B), enhanced ROS production (Fig. 2C, E), and diminished GSH levels (Fig. 2F). Additionally, we observed significant reductions in cell viability (Fig. 2D) and GPX4 expression (Fig. 2G, H). Notably, these alterations were effectively reversed by ferrostatin-1 treatment, establishing a direct link between ferroptotic cell death and microglial activation in response to α-syn. These findings reveal a previously unrecognized mechanism whereby LRRK2 may modulate microglial activation and neuroinflammation through regulation of ferroptotic pathways. This novel insight suggests that targeting ferroptosis might represent a promising therapeutic strategy for managing neuroinflammation in Parkinson’s disease.

### Ferroptosis Mediates NF-κB p65 Activation and Neuroinflammatory Response in Microglia

**Fig. 3:**
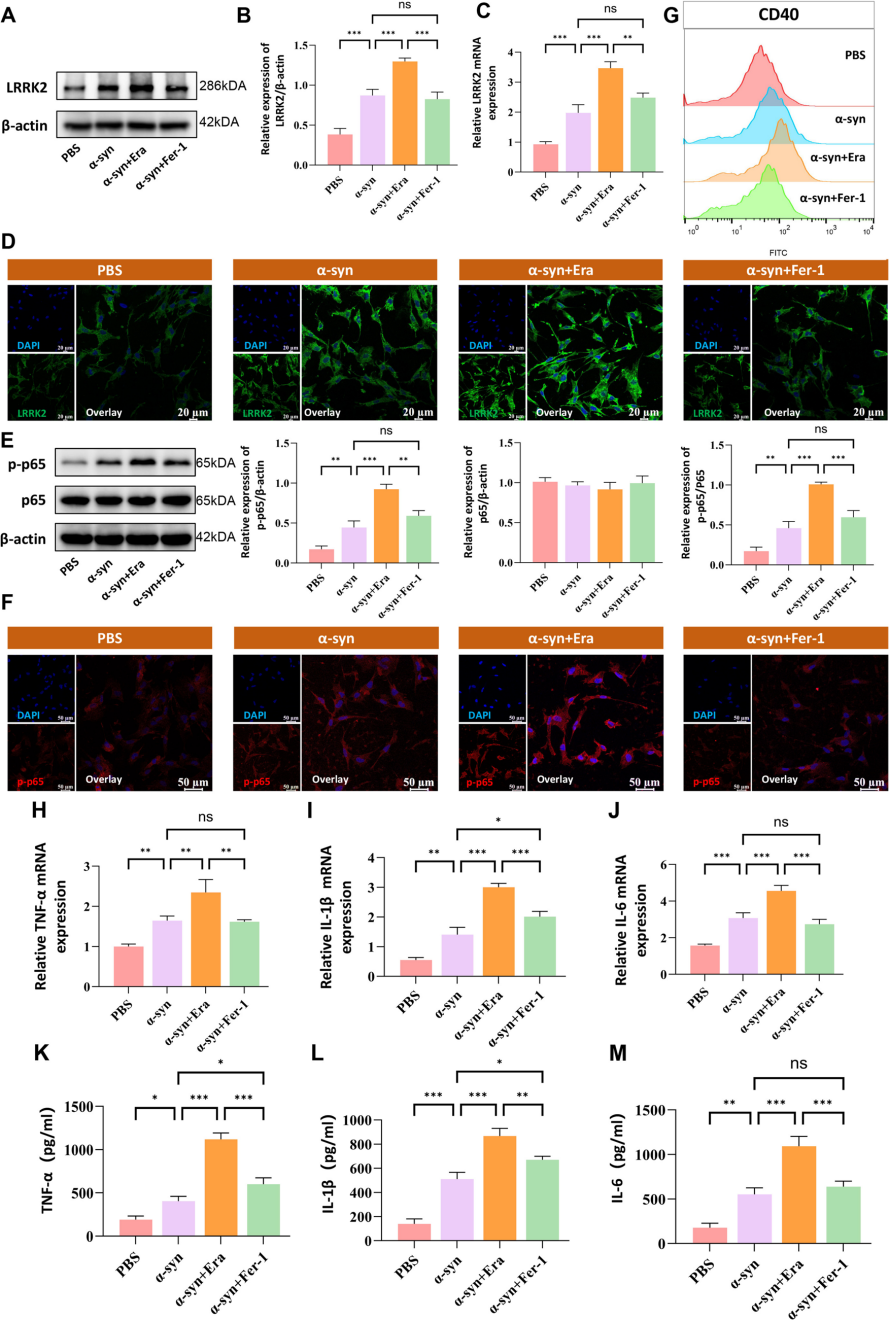
Inhibition of LRRK2 downregulates ferroptosis and p-p65 expression. To investigate the relationship between ferroptosis and inflammatory response, BV2 microglial cells were treated with Erastin (Era,10 μM) or Ferrostatin-1 (Fer-1,1 μM) for 24 h. LRRK2 expression was analyzed by Western blot (**A**), quantified relative to β-actin (**B**), assessed by RT-qPCR (**C**), and further validated by immunofluorescence staining in primary microglia (**D**). NF-κB p65 expression and localization were examined by immunofluorescence (**E**) and Western blot analysis (**F**). CD40 surface expression, a marker of microglial activation, was evaluated by flow cytometry (**G**). Pro-inflammatory cytokine expression was assessed at both mRNA and protein levels by RT-qPCR and ELISA for TNF-α (H, K), IL-1β (I, L), and IL-6 (J, M).Data were presented as means ± SD. The experiments were carried out three times (*n* = 3). One-way analysis of variance (ANOVA) followed by Tukey’s multiple comparison tests in (**B**, **C**, **E**, **H**, **I**, **J**, **K**, **L**, **M**). The difference in folds is statistically significant. *P < 0.05, **P < 0.01, ***P < 0.001. PBS, phosphate‐buffered saline; mRNA, messenger RNA

Given the established role of NF-κB signaling in inflammatory responses, particularly through p65 subunit activation, we investigated its relationship with ferroptosis in microglial cells. Western blot analysis and RT-qPCR revealed that Erastin treatment significantly elevated LRRK2 expression levels (Fig. 3A–C), which was further confirmed by immunofluorescence staining in primary microglia (Fig. 3D). Notably, both immunofluorescence and protein analysis demonstrated enhanced NF-κB p65 expression following Erastin treatment, an effect that was markedly attenuated by Ferrostatin-1 (Fig. 3E, F). Consistent with these findings, CD40 expression, a key marker of microglial activation, was significantly upregulated under Erastin stimulation but reversed by Ferrostatin-1 treatment (Fig. 3G). Furthermore, comprehensive analysis of inflammatory mediators through RT-qPCR and ELISA revealed that ferroptosis inhibition effectively suppressed the production of pro-inflammatory cytokines, including TNF-α (Fig. 3H, K), IL-1β (Fig. 3I, L), and IL-6 (Fig. 3J, M). These data collectively demonstrate that ferroptosis drives microglial activation and neuroinflammation through NF-κB p65 upregulation, while its suppression effectively attenuates this inflammatory cascade.

### LRRK2 Modulates Ferroptosis via the p62-Keap1-Nrf2 Pathway

**Fig. 4:**
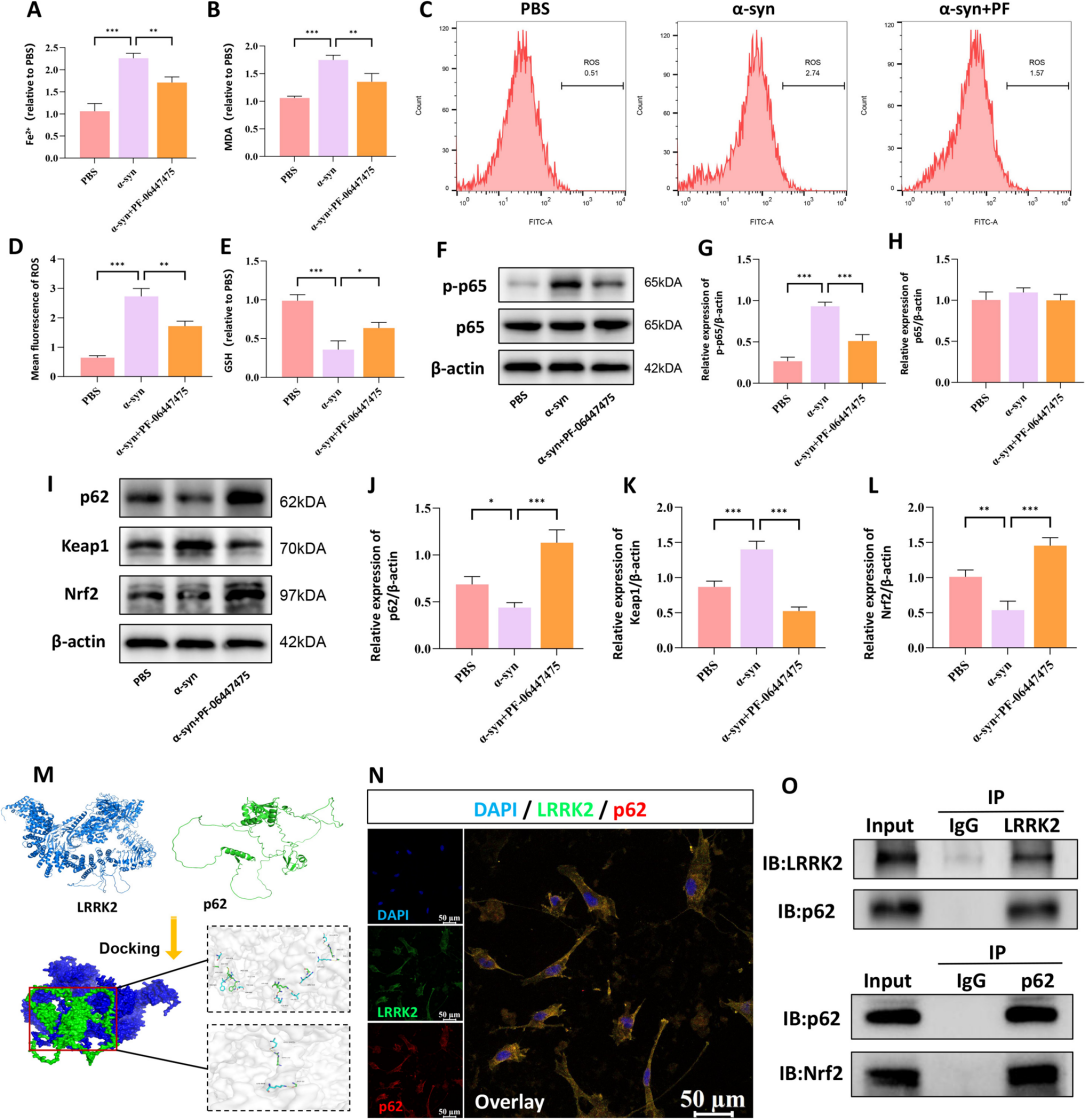
LRRK2 modulates ferroptosis through the p62-Keap1-Nrf2 pathway. BV2 cells were treated with α-synuclein (α-syn, 10 μg/mL) alone or in combination with the LRRK2 inhibitor PF-06447475 (2 μg/mL) for 24 h. Ferroptotic parameters were assessed, including intracellular Fe^2+^ levels (**A**), MDA content (**B**), ROS levels by flow cytometry with quantification (**C**, **D**), and GSH levels (**E**). The expression and phosphorylation of NF-κB pathway components were analyzed by Western blot with quantification of total NF-κB and p-p65 (**F**–**H**). Proteins related to the p62-Keap1-Nrf2 pathway were examined by Western blot with quantification of p62 and Nrf2 (**I**–**L**). Molecular docking analysis using PYMOL was performed to assess the binding affinity between LRRK2 and p62 (**M**), The bule color represents the LRRK2 chain, and the green color represents the p62 chain. Several hydrogen bond interactions are formed between specific amino acids in LRRK2 and specific amino acids in p62, including SER979, GLY204, ARG830, PHE940, MET200, GLU198, TRP198, SER881, LYS435, GLN432, VAL921, GLN923, ARG918, SER407, LYS906, GLU2075, ARG22, GLY12, GLU2075, ARG22, LYS906, and GLY12. These hydrogen bond interactions are crucial for the stable binding ofthe two proteins. And their interaction was visualized by immunofluorescence co-localization (**N**). Antibodies specific for LRRK2 and p62, p62 and Nrf2 (**O**) were used to immunoprecipitation (IP) and reverse-IP lysates from BV2 cells. Western blot analysis ofimmunoprecipitated proteins was performed using antibodies specific for LRRK2 and p62 、p62 and Nrf2. Data were normalized to β-actin. Data were presented as means ± SD. The experiments were carried out three times (*n* = 3). One-way analysis of variance (ANOVA) followed by Tukey’s multiple comparison tests in (**A**, **B**, **D**, **E**, **G**, **H**, **J**, **K**, **L**). The difference in folds is statistically significant. *P < 0.05, **P < 0.01, ***P < 0.001. PBS, phosphate‐buffered saline; MDA, Malondialdehyde; GSH, glutathione; mRNA, messenger RNA

Accumulating evidence suggests that the p62-Keap1-Nrf2 signaling pathway plays a pivotal role in cellular defense against oxidative stress and ferroptosis [34, 35]. These studies demonstrated that p62-mediated sequestration of Keap1 leads to Nrf2 stabilization and transcriptional activation of antioxidant-response genes, thereby mitigating oxidative damage and preventing ferroptotic cell death. However, the interplay between this signaling cascade and LRRK2 in neural cells, as well as the underlying mechanisms, remains elusive. To elucidate the regulatory relationship between LRRK2 and this signaling cascade, we conducted comprehensive analyses in BV2 microglial cells. LRRK2 inhibition by PF-06447475 significantly attenuated multiple ferroptotic indicators, including Fe^2+^ levels (Fig. 4A), MDA content (Fig. 4B), and ROS generation (Fig. 4C, D), while enhancing GSH levels (Fig. 4E). Moreover, PF-06447475 treatment resulted in decreased NF-κB and p-p65 expression (Fig. 4F–H), indicating suppression of inflammatory signaling. Notably, LRRK2 inhibition led to significant upregulation of both p62 and Nrf2 expression (Fig. 4I–L). The direct interaction between LRRK2 and p62 was validated through molecular docking analysis using PYMOL (Fig. 4M) and confirmed by immunofluorescence co-localization studies (Fig. 4N). Additionally, the interactions between LRRK2 and p62, as well as p62 and Nrf2, were validated through co-immunoprecipitation experiments (Fig. 4O). These findings suggest that LRRK2 modulates ferroptosis through negative regulation of the p62-Keap1-Nrf2 pathway.

### Overexpression of p62 Protects BV2 Cells from LRRK2-Induced Ferroptosis and Neuroinflammation

**Fig. 5:**
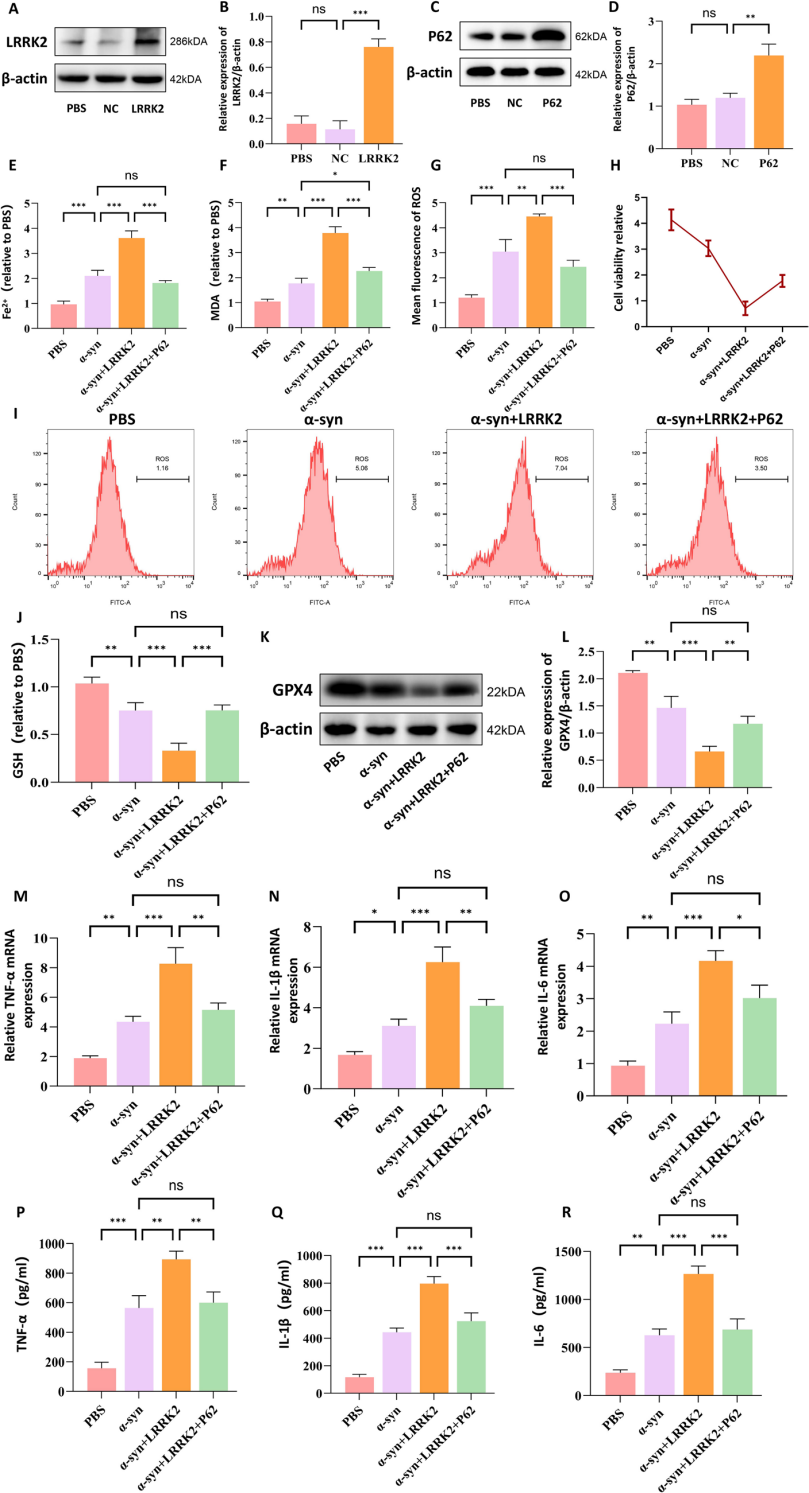
Overexpression of p62 mitigates the ferroptosis and inflammatory response induced by the upregulation of LRRK2 protein. BV2 cells were transfected with LRRK2 or p62 overexpression vectors. Transfection efficiency was confirmed by Western blot analysis of LRRK2 (**A**) and p62 (**C**) with corresponding quantification (**B**, **D**). Ferroptotic markers were measured, including Fe^2+^ levels (**E**), MDA content (**F**), ROS levels by flow cytometry with quantification (**G**, **I**), cell viability (**H**), GSH levels (**J**), and GPX4 expression by Western blot with quantification (**K**, **L**). Pro-inflammatory cytokine expression was assessed at both mRNA and protein levels by RT-qPCR and ELISA for TNF-α (M,P), IL-6 (**N**, **Q**), and IL-1β (**O**, **R**). Data were presented as means ± SD. The experiments were carried out three times (*n* = 3). One-way analysis of variance (ANOVA) followed by Tukey’s multiple comparison tests in (**B**, **D**, **E**, **F**, **G**, **J**, **L**, **M**, **N**, **O**, **P**, **Q**, **R**). The difference in folds is statistically significant. *P < 0.05, **P < 0.01, ***P < 0.001. NC, Negative Control; PBS, phosphate‐buffered saline; MDA, Malondialdehyde; GSH, glutathione; mRNA, messenger RNA

Previous studies have demonstrated that the activation of p62 exerts significant protective effects against ferroptosis[36]. This protection is believed to arise from p62-mediated sequestration of pro-ferroptotic factors and the stabilization of antioxidant signaling pathways. Building upon this finding, our study further investigated the protective role of p62 in LRRK2-induced ferroptosis in BV2 cells. To gain a deeper understanding of the mechanisms underlying the p62-Keap1-Nrf2 pathway in BV2 cells, we constructed overexpression vectors for LRRK2 and p62 and successfully transfected them into BV2 cells. The transfection results showed a significant increase in LRRK2 (Fig. 5A, B)and p62 (Fig. 5C, D)protein levels. Our research revealed that the co-overexpression of p62 and LRRK2 significantly attenuated the changes in ferroptosis-related markers caused by elevated LRRK2 levels. Specifically, we observed a marked reduction in Fe^2+^ (Fig. 5E), MDA (Fig. 5E), and ROS accumulation (Fig. 5G, I), enhanced cell viability (Fig. 5H),a significant increase in GSH levels (Fig. 5J), and a restoration of GPX4 activity (Fig. 5K, L). Furthermore, RT-qPCR analysis and ELISA demonstrated that p62 overexpression reversed the upregulation of pro-inflammatory cytokines induced by LRRK2 elevation, as evidenced by the significant downregulation of TNF-α(Fig. 5M, P), IL-6(Fig. 5N, Q), and IL-1β(Fig. 5O, R) expression levels. These findings indicate that p62 overexpression not only significantly reduces LRRK2-induced ferroptosis but also suppresses the associated neuroinflammatory response.

### LRRK2 Inhibition or p62 Overexpression Confers Neuroprotection Against Microglial-Induced Toxicity

**Fig. 6:**
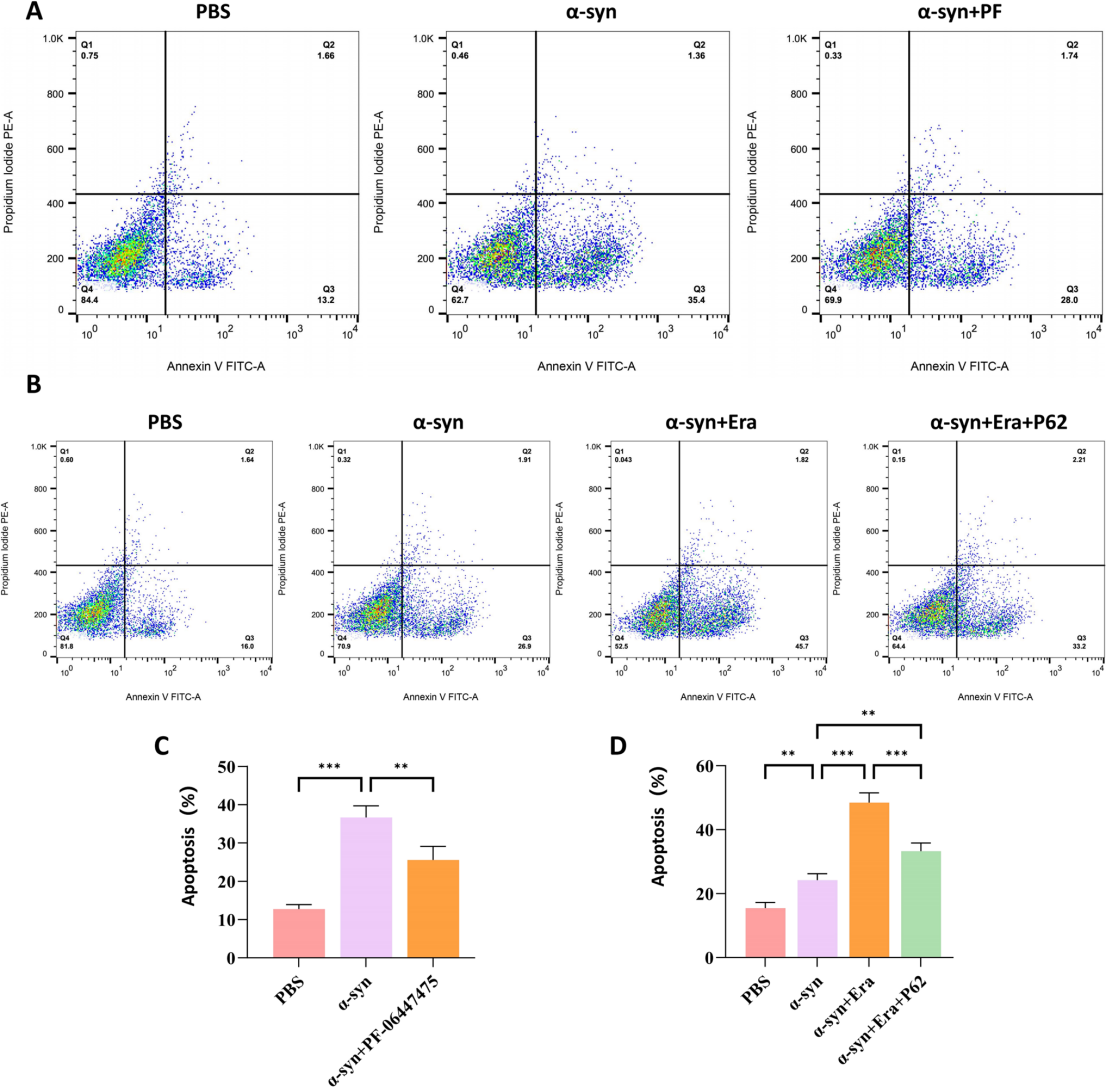
Downregulation of LRRK2 or overexpression of p62 prevented apoptosis following microglial activation in the microglial culture supernatant (MCS) transfer model. SH-SY5Y cells were incubated with conditioned medium from BV2 cells treated with α-synuclein(α-syn,10 μg/mL), PF-06447475(2 μg/mL), or p62 overexpression plasmid. Neuronal apoptosis was assessed by flow cytometric analysis (**A**, **C**). SH-SY5Y cells were incubated with conditioned medium from BV2 cells treated with α-synuclein(α-syn,10 μg/mL), Erastin(Era,10 μM), and p62 overexpression plasmid (**B**, **D**).Data were presented as means ± SD. The experiments were carried out three times (*n* = 3). One-way analysis of variance (ANOVA) followed by Tukey’s multiple comparison tests in (**C**, **D**). The difference in folds is statistically significant. *P < 0.05, **P < 0.01, ***P < 0.001. PBS, phosphate‐buffered saline

Using a conditioned medium transfer model, we evaluated the neuroprotective potential of LRRK2 inhibition and p62 overexpression. BV2 cells were pretreated with either PF-06447475 or transfected with p62 overexpression plasmid prior to α-syn exposure. The conditioned medium was subsequently applied to SH-SY5Y neurons. Both interventions significantly reduced α-syn-induced neuronal apoptosis (Fig. 6A, C). Moreover, while the ferroptosis inducer Erastin potentiated α-syn-mediated neurotoxicity, p62 overexpression effectively protected against this enhanced apoptotic response (Fig. 6B, D). These findings establish that both LRRK2 inhibition and p62 overexpression provide significant neuroprotection against microglial-mediated toxicity, potentially through modulation of the p62-Keap1-Nrf2 pathway and ferroptotic processes.

### Elevated Expression of LRRK2 in MPTP-Induced Parkinson’s Disease Model

**Fig. 7:**
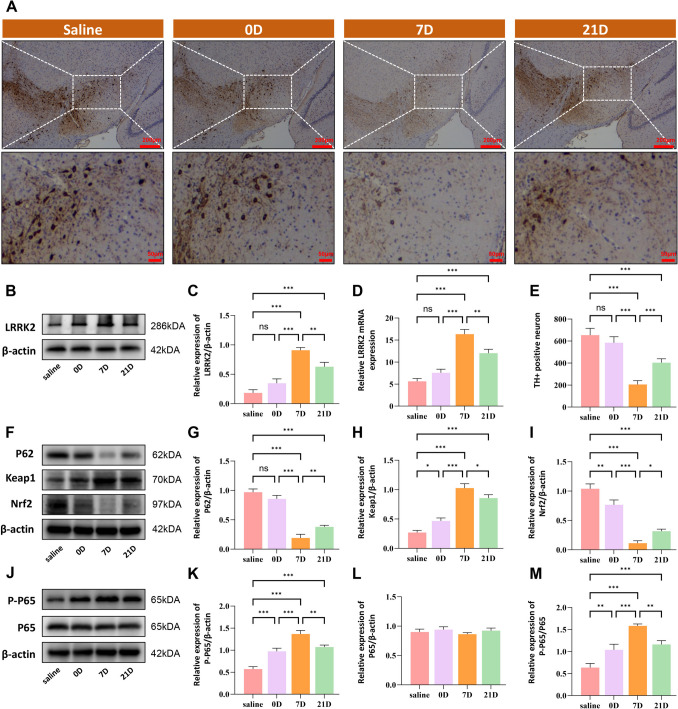
Temporal analysis of LRRK2 expression and associated pathways in MPTP-induced Parkinson’s disease model. 1‐Methyl‐4‐phenyl‐1,2,3,6‐tetrahydropyridine (MPTP) injection can induce mice microglia activation and apoptosis of dopamine (DA). Increased expression levels of LRRK2, p62, and p-p65 were observed in the substantia nigra pars compacta (SNpc) of MPTP‐treated mice in vivo. The mice received daily intraperitoneal injections of MPTP–HCl for 5 consecutive days, while the control mice received saline injections. Subsequently, the mice were decapitated, and midbrains were harvested at different time points following MPTP intoxication, including immediately after the last MPTP injection, as well as at 0, 7, and 21 days postinjection. Representative images of TH immunohistochemistry in SNpc showing progressive loss of dopaminergic neurons at indicated time points post-MPTP injection (**A**, **E**). Scale bar: 200 µm and 50 µm. Western blot and RT-qPCR analysis demonstrated significant upregulation of LRRK2 levels (**B**–**D**). Western blot analysis showed dynamic changes in key proteins (**F**), with quantification revealing altered expression of p62 (**G**), Keap1 (**H**), and Nrf2 (**I**). Additional Western blot analysis (**J**) with quantification demonstrated changes in p-p65 (**K**), p65 (**L**), and p-p65/p65 ratio (M). Data were presented as means ± SD. The experiments were carried out three times (*n* = 3). One-way analysis of variance (ANOVA) followed by Tukey’s multiple comparison tests in (**C**, **D**, **E**, **G**, **H**, **I**, **K**, **L**, **M**). The difference in folds is statistically significant. *P < 0.05, **P < 0.01, ***P < 0.001. mRNA, messenger RNA

To elucidate the mechanistic role of LRRK2 in PD pathogenesis, we established an MPTP-induced mouse model of PD and conducted temporal analysis of LRRK2 expression in the midbrain region. Experimental time points included baseline (saline control) and post-MPTP administration at days 0, 7, and 21. Initial characterization using tyrosine hydroxylase (TH) immunohistochemistry confirmed substantial dopaminergic neuronal loss following MPTP treatment (Fig. 7A, E). Subsequent molecular analyses revealed a significant temporal elevation in LRRK2 expression, as evidenced by both Western blot and qRT-PCR analyses (Fig. 7A–C).To comprehensively understand the molecular interplay between LRRK2 and its downstream effectors, we examined the expression profiles of p62 and Nrf2 using Western blot analysis (Fig. 7F–I). Notably, both p62 and Nrf2 exhibited biphasic expression patterns, with significant downregulation at day 7 post-MPTP treatment followed by partial recovery by day 21. Concomitantly, we observed marked upregulation of p-p65 protein levels in the MPTP-induced PD model (Fig. 7J–M). These findings suggest that LRRK2, p62, and P‐p65 may serve as neuroinflammation markers in the pathogenesis of PD.

### Concurrent Alterations in p62, LRRK2 Expression and Microglial Activation in MPTP-Induced Parkinson's Disease Model

**Fig. 8:**
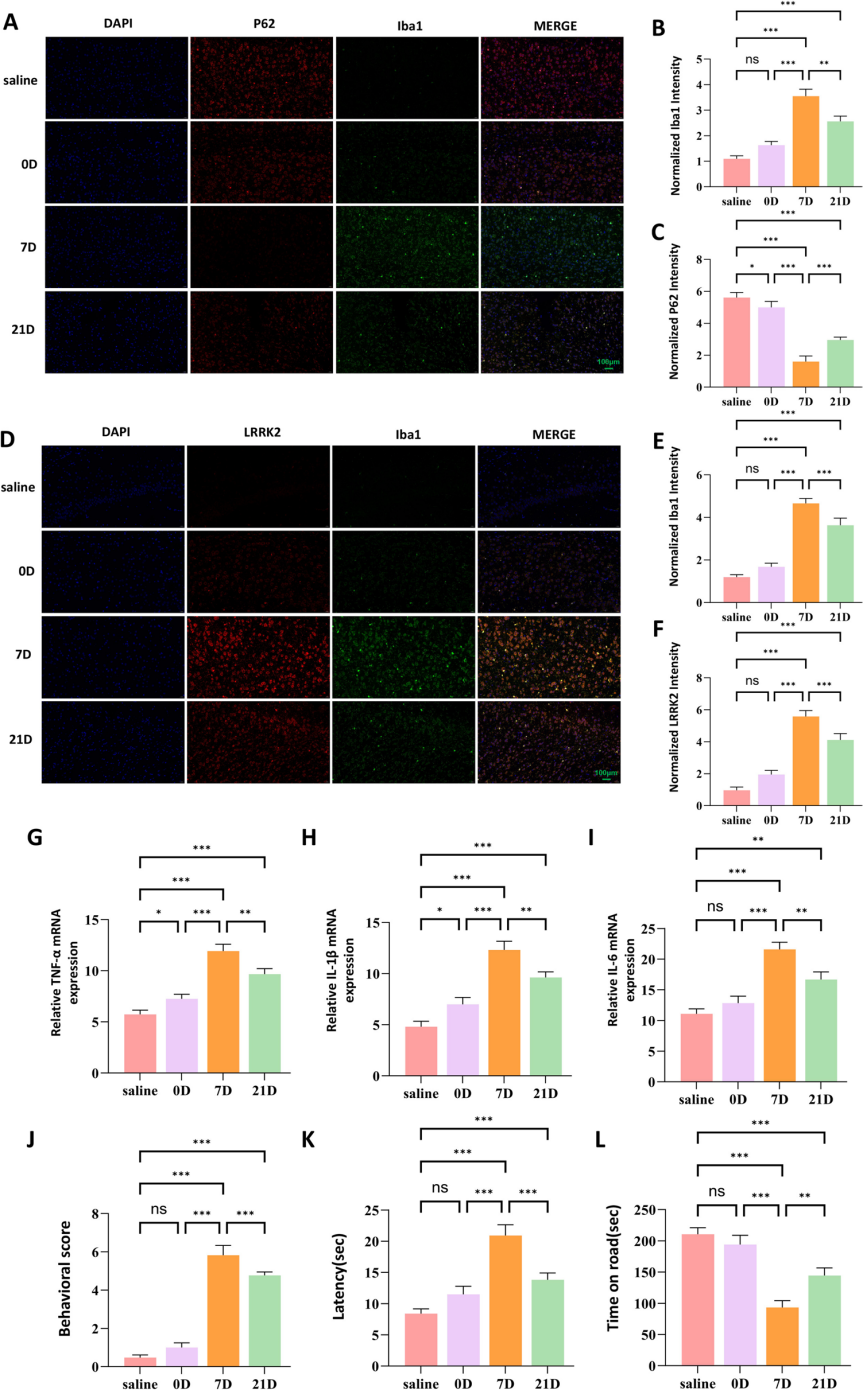
Concurrent Alterations in p62, LRRK2, and Microglial Activation in MPTP-Induced PD Model.Immunofluorescence analysis of the substantia nigra pars compacta (SNpc) showing increased Iba1 expression (green) accompanied by reduced p62 expression (red) in MPTP-treated mice compared to the saline control (**A**). Quantification of Iba1 and p62 immunofluorescence intensity is presented in (**B**) and (**C**), respectively.Immunofluorescence analysis of the SNpc demonstrating increased Iba1 expression (green) when LRRK2 expression (red) is upregulated in MPTP-treated mice compared to the saline control (**D**). Quantification of Iba1 and LRRK2 immunofluorescence intensity is shown in (**E**) and (**F**), respectively.Quantitative RT-PCR analysis of the pro-inflammatory cytokines TNF-α, IL-1β, and IL-6 in the midbrain of saline and MPTP-treated mice (**G**–**I**).Behavioral assessments of motor function in saline and MPTP-treated mice, including the behavioral score (J), rod climbing test (**K**), and rotarod test (**L**). Data were presented as means ± SD. The experiments were carried out three times (*n* = 3). One-way analysis of variance (ANOVA) followed by Tukey’s multiple comparison tests in (**B**, **C**, **E**, **F**, **G**, **H**, **I**, **J**, **K**, **L**). The difference in folds is statistically significant. *P < 0.05, **P < 0.01, ***P < 0.001. mRNA, messenger RNA

To further delineate the role of p62 in PD pathogenesis, we employed immunofluorescence techniques to analyze its expression patterns in the MPTP model. Our analyses revealed an inverse correlation between p62 expression and microglial activation, as evidenced by the microglial marker Iba1. Specifically, regions exhibiting elevated p62 expression showed corresponding reductions in Iba1 immunoreactivity (Fig. 8A–C). Conversely, areas of increased LRRK2 expression demonstrated diminished p62 fluorescence intensity coupled with enhanced Iba1 expression (Fig. 8D–F). Molecular profiling of inflammatory mediators revealed significant upregulation of pro-inflammatory cytokines, including TNF-α, IL-1β, and IL-6 (Fig. 8G–I) in MPTP-treated mice. To establish the functional consequences of these molecular alterations, we conducted comprehensive behavioral assessments. MPTP-treated mice exhibited significant impairments in motor function compared to controls (Fig. 8J–L). These findings collectively suggest that microglial activation and associated neuroinflammatory responses are integral components of MPTP-induced neurodegeneration, potentially representing viable therapeutic targets.

### Inhibition of LRRK2 Enhances p62 Expression in MPTP-Treated Mice

**Fig. 9:**
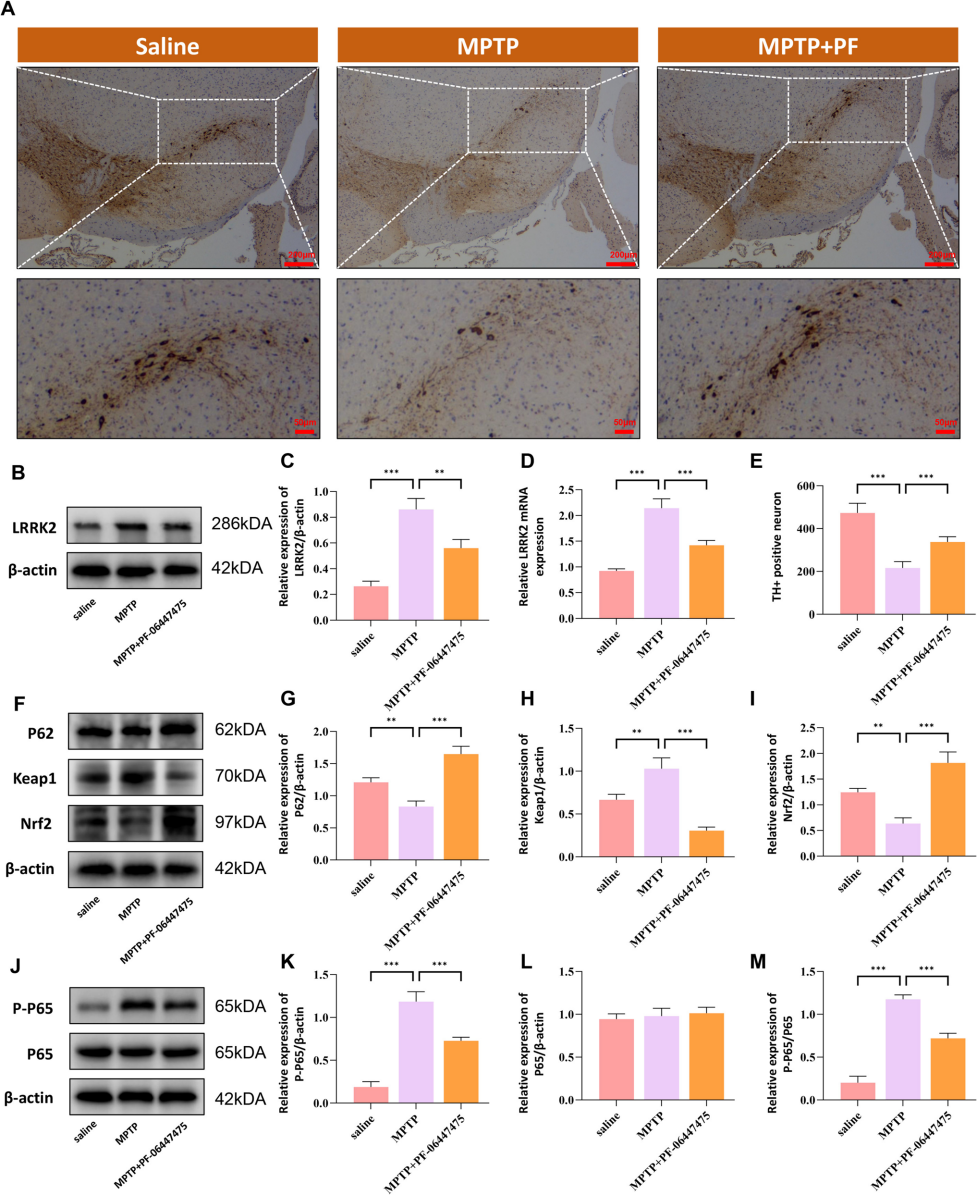
Inhibition of LRRK2 Attenuates Dopaminergic Neuronal Damage and Modulates Microglial Activation in MPTP-Treated Mice.Mice were orally administered PF‐06447475 via gastric feeding for a duration of 14 days. Subsequently, the mice received daily intraperitoneal injections of MPTP–HCl for a total of 5 consecutive days, while the control mice were subjected to saline injections. The treatment with PF‐06447475 was initiated 2 days before the MPTP injection, and the midbrain samples were collected 7 days after the final MPTP injection.Immunohistochemical staining for TH in the substantia nigra pars compacta of saline, MPTP-treated, and MPTP + LRRK2 inhibitor (PF-06447475)-treated mice (**A**). Scale bar: 200 µm and 50 µm. Quantification of TH-positive neurons is shown in (**E**). Western blot and quantitative RT-PCR analyses of LRRK2 expression in the substantia nigra pars compacta of saline, MPTP-treated, and MPTP + LRRK2 inhibitor-treated mice(**B**–**D**).Western blot analyses of p62, Keap1, and Nrf2 expression in the substantia nigra pars compacta of saline, MPTP-treated, and MPTP + LRRK2 inhibitor-treated mice (**F**–**I**).Western blot analyses of phosphorylated NF-κB p65 (p-p65) expression in the substantia nigra pars compacta of saline, MPTP-treated, and MPTP + LRRK2 inhibitor-treated mice (**J**–**M**). Data were presented as means ± SD. The experiments were carried out three times (*n* = 3). One-way analysis of variance (ANOVA) followed by Tukey’s multiple comparison tests in (**C**, **D**, **E**, **G**, **H**, **I**, **K**, **L**, **M**). The difference in folds is statistically significant. *P < 0.05, **P < 0.01, ***P < 0.001. mRNA, messenger RNA

Building upon our in vitro findings demonstrating LRRK2-mediated regulation of ferroptosis through the p62-Keap1-Nrf2 pathway, we investigated the therapeutic potential of LRRK2 inhibition in vivo. MPTP-treated mice received oral administration of the LRRK2 inhibitor PF-06447475. TH immunohistochemistry revealed significant preservation of dopaminergic neurons following LRRK2 inhibition (Fig. 9A, E). Molecular analyses demonstrated that PF-06447475 treatment effectively reduced LRRK2 expression (Fig. 9B–D) while simultaneously increasing p62 and Nrf2 levels in the SNpc (Fig. 9F–I). Furthermore, LRRK2 inhibition resulted in marked reduction of p-p65 expression (Fig. 9J–M), indicating suppression of NF-κB signaling. These results demonstrate that LRRK2 inhibition provides neuroprotection through dual mechanisms: upregulation of the cytoprotective p62-Keap1-Nrf2 pathway and attenuation of NF-κB-mediated inflammatory signaling.

### LRRK2 Inhibition Ameliorates Neuroinflammation and Motor Deficits in MPTP-Induced Mice

**Fig. 10:**
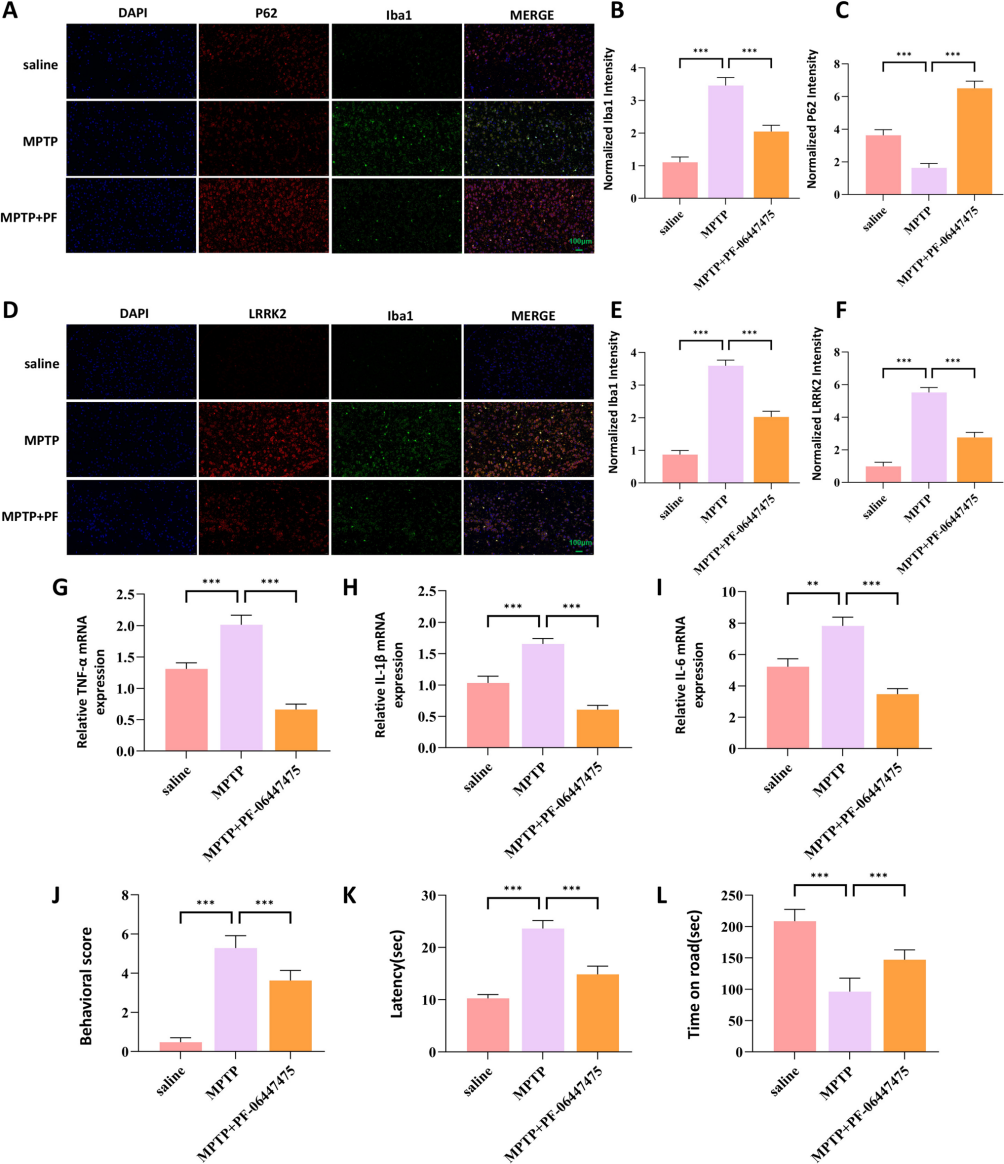
Inhibition of LRRK2 Reduces Microglial Activation and Enhances p62 Expression in MPTP-Induced Parkinson’s Disease Mice.Immunofluorescence analysis of the substantia nigra pars compacta (SNpc) showing p62 (red) and Iba1 (green) expression in saline, MPTP-treated, and MPTP + PF-06447475-treated mice (**A**). Quantification of p62 and Iba1-positive cells is presented in (**B**) and (**C**), respectively.Immunofluorescence analysis of the SNpc illustrating LRRK2 (red) and Iba1 (green) expression in saline, MPTP-treated, and MPTP + PF-06447475-treated mice (D). Quantification of LRRK2 and Iba1-positive cells is shown in (**E**) and (**F**), respectively.Quantitative RT-PCR analysis of the pro-inflammatory cytokines IL-6, TNF-α, and IL-1β in the substantia nigra pars compacta of saline, MPTP-treated, and MPTP + PF-06447475-treated mice (**G**–**I**).Behavioral assessments of motor function in control, MPTP-treated, and MPTP + PF-06447475-treated mice, including the behavioral score (**J**), rod climbing test (**K**), and rotarod test (**L**).Data were presented as means ± SD. The experiments were carried out three times (*n* = 3). One-way analysis of variance (ANOVA) followed by Tukey’s multiple comparison tests in (**B**, **C**, **E**, **F**, **G**, **H**, **I**, **J**, **K**, **L**). The difference in folds is statistically significant. *P < 0.05, **P < 0.01, ***P < 0.001. mRNA, messenger RNA

Immunofluorescence analysis of PF-06447475-treated mice revealed significant upregulation of p62 expression concurrent with reduced Iba1 immunoreactivity (Fig. 10A–C). The reduction in LRRK2 expression following inhibitor treatment was accompanied by diminished microglial activation, as evidenced by decreased Iba1 levels (Fig. 10D–F). Moreover, PF-06447475 treatment significantly attenuated the expression of pro-inflammatory cytokines, including TNF-α, IL-1β, and IL-6 (Fig. 10G–I). To assess the functional implications of LRRK2 inhibition, we performed comprehensive behavioral analyses. PF-06447475-treated mice demonstrated significant improvement in motor function compared to MPTP-treated controls (Fig. 10J–L). These findings provide compelling evidence that LRRK2 inhibition confers neuroprotection in the MPTP model through multiple mechanisms: enhancement of the p62-Keap1-Nrf2 pathway, suppression of microglial activation, and attenuation of neuroinflammatory responses.

## Discussion

PD is a prevalent neurodegenerative disorder primarily affecting the elderly population. Despite extensive research efforts, the complex pathogenesis of PD remains elusive. The accumulation of α-syn fibrils is a hallmark pathological feature of PD. As the disease progresses, pathological α-syn fibrils can propagate and spread between neurons via membrane receptors on the neuronal surface [37, 38]. Furthermore, studies have revealed that α-syn aggregates can activate microglia, leading to the release of pro-inflammatory cytokines such as TNF-α and IL-1β, which can subsequently cause neuronal damage [39]. Activated microglia can induce oxidative stress in dopaminergic neurons, ultimately resulting in neuronal death. However, the precise mechanisms by which α-syn triggers microglial activation and the consequent dopaminergic neuronal apoptosis remain unclear. Mounting evidence suggests that microglial activation plays a pivotal role in the inflammatory pathogenesis of PD and correlates with the stage and severity of the disease [40, 41]. Traditional neuroinflammation models have predominantly relied on LPS stimulation to induce cellular neuroinflammation. Although LPS is a potent activator of innate immunity and has been extensively employed to probe microglial responses, it largely reflects a generic bacterial inflammatory stimulus rather than the central pathology of PD [42]. To address this limitation, we developed a more pathologically relevant model by stimulating BV2 microglial cells with α-syn, which better recapitulates the disease-specific features of PD compared with conventional LPS-driven paradigms. α-syn, a hallmark protein in PD, can bind directly to surface receptors such as TLR2, thereby triggering microglial activation and cytokine release [43, 44]. Consequently, α-syn–based stimulation more accurately mirrors pathogenic processes implicated in PD progression. In contrast, LPS-induced neuroinflammation heavily involves TLR4/MyD88-dependent pathways, which, despite eliciting robust inflammatory responses, do not capture key aspects of PD pathology. Importantly, α-syn–dependent microglial activation encompasses not only inflammatory signaling but also oxidative damage and ferroptotic cell death—both core hallmarks of PD. Extensive evidence shows that chronic α-syn accumulation primes and activates microglia in the aging or diseased brain, perpetuating a harmful cycle of inflammation and neurodegeneration [45]. By employing α-syn to provoke microglial responses, our model recapitulates critical elements of PD pathology, including progressive protein aggregation, microglial stress, and neuronal vulnerability. Through this physiologically relevant framework, we achieve a closer approximation of PD’s underlying neuroinflammatory processes, enabling more accurate investigations into disease progression and potential therapeutic interventions.In our study, we observed upregulated expression of the microglial activation marker CD40 in α-syn-stimulated BV2 microglial cells, accompanied by significantly elevated levels of pro-inflammatory cytokines TNF-α, IL-1β, and IL-6, indicating successful activation of BV2 microglia by α-syn. Furthermore, dopaminergic neuronal apoptosis experiments demonstrated that α-syn-induced neuroinflammation can lead to increased neuronal cell apoptosis. Thus, our PD neuroinflammation model, constructed by stimulating BV2 cells with α-syn protein, more accurately reflects PD pathology compared to the traditional LPS stimulation model. This approach provides a more authentic and reliable experimental basis for subsequent research, enhancing the credibility and translational potential of the findings.

Mutations in the LRRK2 gene, located at the Park8 locus on chromosome 12q12, represent the most prevalent genetic cause of PD, accounting for a significant proportion of familial PD cases. LRRK2, a multi-domain protein, exhibits dual enzymatic functions as both a kinase and GTPase [46]. Enhanced kinase activity of LRRK2 is believed to be one of the key pathological mechanisms implicated in PD, though the precise molecular pathways remain unclear. Historically, research has primarily concentrated on the neuronal role of LRRK2. Studies have demonstrated that overexpression of LRRK2 in primary cultured neurons induces neurotoxicity, characterized by axonal shortening, cell death, or impaired organelle function [47, 48]. In rodent models, LRRK2 expression in the striatum of mice or rats leads to pronounced degeneration of dopaminergic neurons within the SNpc [49, 50]. Notably, LRRK2 is highly expressed in microglia within the central nervous system. A study published in Lancet Neurology reported that PD patients carrying LRRK2 mutations exhibit significantly increased cholinesterase activity in the cortex, limbic system, and thalamus compared to sporadic PD cases [51]. This cholinergic system alteration may be linked to neuroinflammatory responses within microglia. These findings suggest a complex and indirect role for LRRK2 in the neuroinflammation associated with PD pathology, necessitating further investigation into the underlying mechanisms. Exploring the relationship between LRRK2 and microglia-mediated neuroinflammation is crucial for elucidating the function of LRRK2 in PD.Our research identified a marked upregulation of LRRK2 expression in BV2 microglial cells following stimulation with α-syn, with expression levels increasing in a dose- and time-dependent manner. This upregulation correlated with microglial activation and elevated levels of pro-inflammatory cytokines IL-6, TNF-α, and IL-1β. Subsequent experiments revealed that inhibiting LRRK2 expression with PF-06447475 attenuated α-syn-induced microglial activation and pro-inflammatory cytokine expression, concomitantly reducing neuronal apoptosis. These data underscore the significance of LRRK2 in mediating microglial neuroinflammation and DA neuronal apoptosis in PD pathology.Furthermore, in an MPTP-induced PD mouse model, elevated LRRK2 expression was associated with microglial activation and increased levels of pro-inflammatory cytokines. Treatment with PF-06447475 effectively suppressed microglial activation and cytokine expression. Both in vivo and in vitro findings underscore the therapeutic potential of LRRK2 inhibition in mitigating microglial activation.

NF-κB represents a widely distributed family of transcription factors that governs both innate and adaptive immunity in the central nervous system [52]. Substantial evidence indicates that α-syn pathology in PD can potentiate NF-κB activation in microglia, thereby intensifying neuroinflammation and oxidative stress [53]. The p65 subunit of NF-κB is constitutively expressed, but its phosphorylation at specific serine residues (e.g., Ser536) is crucial for transcriptional activation and nuclear translocation [54, 55]. Because total p65 levels often remain stable, p-p65 serves as a more sensitive indicator of dynamic NF-κB activity. Our data reveal that aberrant LRRK2 expression in α-syn–stimulated BV2 cells drives a pro-ferroptotic cascade culminating in elevated p-p65 levels and increased microglial activation. Notably, pharmacological inhibition of ferroptosis with ferrostatin-1 significantly reduces p-p65 and dampens microglial inflammatory responses, underscoring ferroptosis as a central mediator of NF-κB–driven neuroinflammation. In parallel, suppressing LRRK2 kinase function via PF-06447475 restricts p-p65 levels and pro-inflammatory cytokine release, reinforcing the importance of p65 phosphorylation in chronic neurodegenerative contexts. LRRK2, as a large multidomain protein with both kinase and GTPase functions, can modulate multiple downstream effectors depending on the cellular context and extracellular stimuli. For instance, LRRK2 expression in microglia has been associated with altered NF-κB transcriptional activity [20, 56], while other studies highlight the importance of NFATC2 as an alternative route by which LRRK2 may influence neuroinflammation [57]. Therefore, LRRK2’s regulatory role over different pathways is not necessarily mutually exclusive but reflects the complexity and versatility of LRRK2-mediated signaling. Although some reports highlight a distinct regulatory axis between LRRK2 and NF-κB signaling in microglia, others point to overlapping mechanisms where LRRK2-dependent phosphorylation of NF-κB subunits (e.g., p50) or associated kinases augments inflammatory outputs [58]. Our data demonstrate that LRRK2 inhibition not only diminishes the release of key pro-inflammatory cytokines but also reduces ferroptosis markers and NF-κB phosphorylation. We propose that simultaneous modulation of both NF-κB and NRF2 pathways by LRRK2 may reflect a broader range of microglial adaptations to α-syn–induced injury. As such, it is foreseeable that LRRK2 orchestrates NF-κB activation while concurrently attenuating NRF2’s antioxidant response, thereby amplifying inflammatory damage in α-syn–induced neurodegeneration.

To further elucidate the specific molecular mechanisms underlying LRRK2-mediated regulation of neuroinflammation, we conducted an in-depth investigation of the p62-Keap1-Nrf2 signaling pathway. p62, a multivalent protein, is involved in the regulation of oxidative stress defense and cellular metabolism [59]. It serves as a key modulator and activator of the Keap1-Nrf2 and nuclear factor-κB signaling pathways, linking p62 to oxidative defense systems and inflammation, respectively [60]. Activation of the p62-Keap1-Nrf2 signaling axis has been reported to effectively suppress ROS accumulation and oxidative stress, thereby attenuating ferroptosis [61, 62]. Nrf2, a crucial transcription factor regulating redox homeostasis, resides in the cytoplasm under non-activated conditions, interacting with Keap1 and undergoing rapid degradation via the ubiquitin–proteasome pathway. This process is tightly controlled by p62, whose elevation directly inhibits Keap1, preventing Nrf2 ubiquitination and leading to nuclear accumulation of Nrf2. Consequently, Nrf2 induces the expression of genes associated with antioxidants, metabolism, and detoxifying enzymes, ultimately inhibiting ferroptosis [63]. Our study revealed that exposure to α-syn and the ferroptosis inducer erastin elevated the expression levels of LRRK2 and p65 in BV2 cells, accompanied by an increase in ferroptosis markers such as GPX4 decrease, Fe2 + , and ROS. Conversely, ferroptosis inhibitors reversed these alterations. The abnormal upregulation of LRRK2 has been identified as a significant trigger for microglial activation and neuroinflammatory responses, with the p62-Keap1-Nrf2 pathway playing a pivotal intermediary role. Our research further explored whether LRRK2 regulates ferroptosis through the p62-Keap1-Nrf2 pathway. By inhibiting LRRK2 using PF-06447475, we observed a significant increase in p62 and Nrf2 levels, concurrent with a marked reduction in intracellular Fe2 + , MDA, ROS, and pro-inflammatory cytokines IL-6, TNF-α, and IL-1β. These findings clearly indicate a potential association between the p62-Keap1-Nrf2 pathway and microglial activation, oxidative stress responses, and neuroinflammation. Moreover, the upregulation of the p62-Keap1-Nrf2 signaling pathway upon LRRK2 inhibition further confirmed that LRRK2 exerts its effects through this pathway.In subsequent experiments, co-expression of p62 and LRRK2 alleviated α-syn-induced microglial activation, oxidative stress responses, and inflammatory cytokine release. Molecular docking visualization studies using PyMOL revealed a significant interaction between LRRK2 and p62, further substantiating the notion that LRRK2 may activate microglia and trigger neuroinflammatory responses by downregulating the p62-Keap1-Nrf2 pathway, ultimately leading to the apoptosis of dopaminergic neurons. Leveraging the MPTP mouse model, we conducted longitudinal analyses of dopaminergic neuron survival and microglial activation at 0, 7, and 21 days post-injection, thereby capturing the progressive neurodegenerative trajectory characteristic of this toxin-based Parkinson’s disease paradigm [64, 65]. This temporal framework enables a detailed examination of how LRRK2-driven ferroptosis and microglial neuroinflammation unfold from acute to more protracted phases of injury, providing a rationale for targeted therapeutic strategies—such as the LRRK2 inhibitor PF-06447475—and highlighting optimal intervention windows to curb ongoing neurodegeneration. Mechanistically, we show that LRRK2 inhibition activates the p62–Keap1–Nrf2 pathway, strengthens antioxidant capacity, and attenuates inflammatory signaling by Iba1 + microglia, underscoring LRRK2’s role in repressing this critical cytoprotective axis. Collectively, these findings pinpoint LRRK2 as a central regulator of ferroptosis and neuroinflammation in Parkinson’s disease, offering compelling evidence for its therapeutic potential and underscoring its broader significance in neurodegenerative pathophysiology.However, it is important to acknowledge the limitations of the current study. Although we observed significant biochemical and cellular effects by inhibiting LRRK2 using PF-06447475, these findings are primarily based on in vitro cell experiments, and their applicability and effectiveness in vivo require further validation. Moreover, this study did not explore the potential compensatory signaling pathways in response to LRRK2 inhibition, and future research should consider comprehensive signaling pathway analyses to fully elucidate the mechanisms of LRRK2 function.

Additionally, we validated the regulatory effects of LRRK2 inhibition on neuroinflammation and oxidative stress using the MPTP mouse model. While MPTP predominantly causes acute dopaminergic neuronal loss by inhibiting mitochondrial complex I, it also induces robust microglial activation and the formation of an inflammatory milieu in the substantia nigra [8, 66]. Our findings thus highlight the therapeutic potential of LRRK2 inhibition in reducing neuroinflammation and subsequent neuronal loss in this model. Nonetheless, we acknowledge that α-syn–based models may more closely approximate the chronic and progressive nature of PD, where α-syn aggregation and gradual neurodegeneration are key hallmarks. Moving forward, validating whether LRRK2 inhibition can similarly dampen microglial activation in α-syn–driven models will be critical for corroborating our therapeutic strategy.the simplicity of these models limits their ability to recapitulate the complexity of human Parkinson’s disease.

Despite these limitations, the present study provides important new insights into the role of LRRK2 in modulating neuroinflammation and ferroptosis through the p62-Keap1-Nrf2 pathway. Previous studies have shown that targeting LRRK2 can upregulate p62 and Nrf2 in microglia, thereby enhancing autophagy and antioxidant responses [67, 68]. Building upon these findings, our work extends to the ferroptotic pathways central to α-syn–induced microglial inflammation. The neuroinflammation model established by stimulating BV2 microglia with α-syn protein more accurately mimics the pathological characteristics of PD neuroinflammation, enhancing the clinical relevance and translational potential of the experimental results. By delineating how ferroptosis markers—Fe^2*^, MDA, GSH, and GPX4—are regulated in tandem with p62/Nrf2 activation, we demonstrate a previously unrecognized convergence between microglial inflammatory responses and ferroptotic vulnerabilities in the context of PD. Furthermore, through molecular docking and mouse model studies, we have unraveled the interactions between LRRK2 and the p62-Keap1-Nrf2 pathway, laying a solid foundation for understanding the pathological mechanisms of Parkinson’s disease and developing targeted therapeutic strategies for LRRK2. This integrated perspective underscores why targeting LRRK2 in microglia has significant therapeutic promise, beyond its known canonical effects on p62 and Nrf2. In conclusion, LRRK2 plays a pivotal role in the pathological processes of neuroinflammation and ferroptosis in Parkinson’s disease. By elucidating its regulatory mechanisms on the p62-Keap1-Nrf2 pathway, we provide new theoretical underpinnings for understanding and intervening in PD. Future research should focus on further validating these mechanisms in preclinical models and human patients, which will contribute to advancing the development of effective treatments for Parkinson’s disease.

## Conclusion

**Fig. 11:**
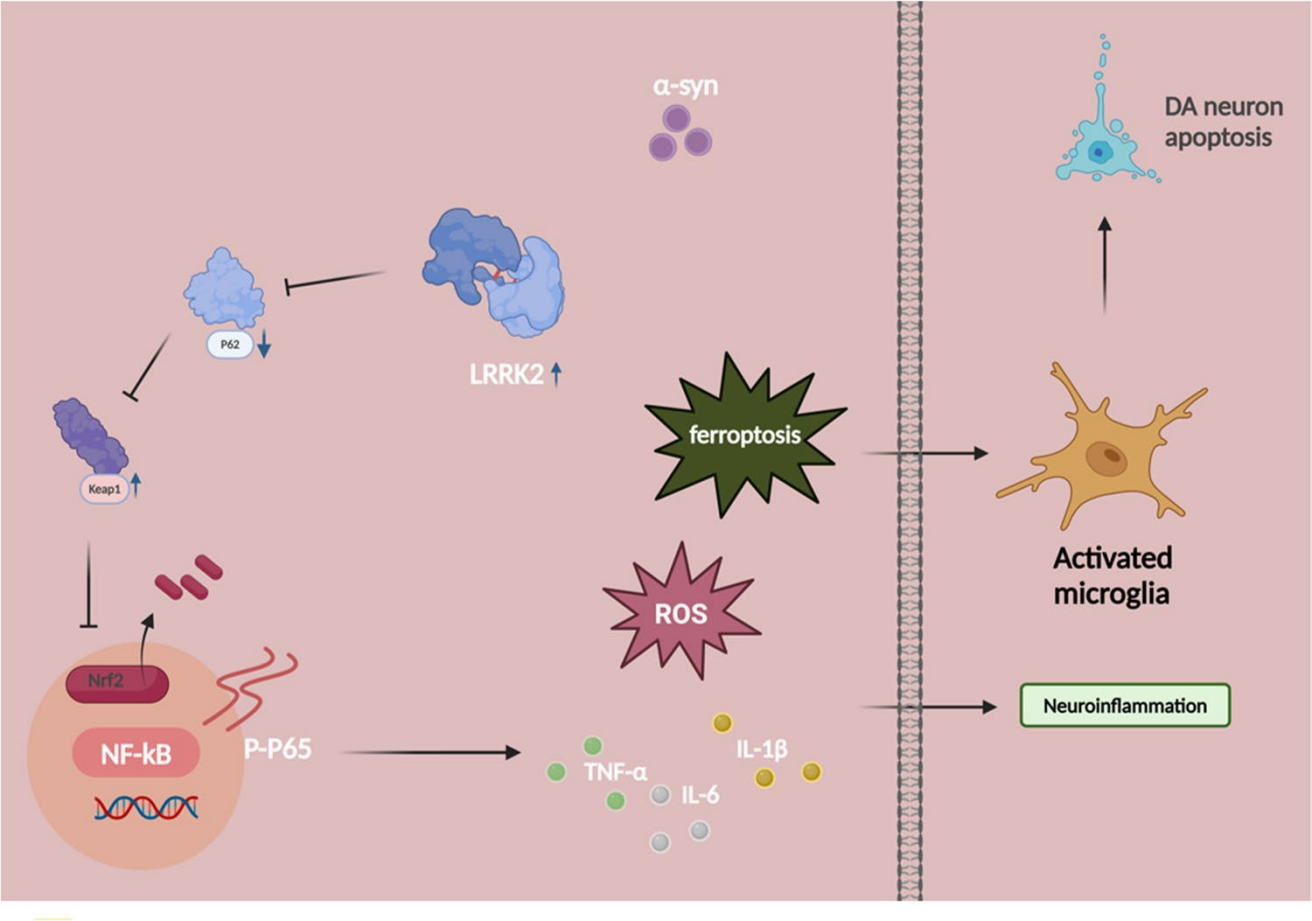
The schematic diagram illustrates the proposed mechanisms by which LRRK2 modulates ferroptosis through the p62-Keap1-Nrf2 signaling pathway, contributing to the inflammatory pathogenesis of PD. This schematic representation provides a comprehensive overview of the key molecular players and their interactions, shedding light on the potential underlying mechanisms driving the pathological processes in PD

This comprehensive study has elucidated the pivotal role of LRRK2 in the pathogenesis of PD by modulating microglial-mediated neuroinflammation and ferroptosis through the p62-Keap1-Nrf2 signaling pathway. In summary, utilizing an α-syn protein-induced microglial model and an MPTP-induced PD mouse model, this study substantiated the correlation between LRRK2 overexpression and the activation of the ferroptosis signaling pathway, which directly contributes to microglial hyperactivation and the apoptosis of midbrain dopaminergic neurons. Inhibition of LRRK2 not only significantly attenuated α-syn-induced microglial inflammatory responses but also conferred neuroprotective effects by alleviating ferroptosis through the activation of the p62-Keap1-Nrf2 signaling pathway. These findings were corroborated in the MPTP-induced PD mouse model, where LRRK2 inhibition effectively attenuated microglial activation, reduced dopaminergic neuronal loss, and enhanced the activity of the p62-Keap1-Nrf2 pathway, leading to improved motor function. In conclusion, this study evidence that LRRK2 plays a central role in the pathogenesis of Parkinson’s disease by orchestrating microglial neuroinflammation and ferroptosis through the p62-Keap1-Nrf2 signaling cascade. These findings not only advance our understanding of the underlying molecular mechanisms driving PD but also highlight the therapeutic potential of targeting LRRK2 and the p62-Keap1-Nrf2 pathway for the development of novel neuroprotective strategies (Fig. 11).

## Supplementary Information

Below is the link to the electronic supplementary material.

Fig. 12 Supplementary file 1

High resolution image (68.7 MB)

Fig. 13 Supplementary file 2

High resolution image (41.1 MB)

Fig. 14 Supplementary file 3

High resolution image (33.3 MB)

ESM 4 (DOCX 13.4 KB)

## Data Availability

The raw data supporting the conclusions of this article will be made available by the authors upon reasonable request. Data is provided within the manuscript or supplementary information files.
