# Supplementary figures and images for: LRRK2 Mediates α-Synuclein-Induced Neuroinflammation and Ferroptosis through the p62-Keap1-Nrf2 Pathway in Parkinson’s Disease
Source: Inflammation. 2025 Apr 2;48(5):3666–91. doi: 10.1007/s10753-025-02291-8 (PMC12596413; doi:10.1007/s10753-025-02291-8)

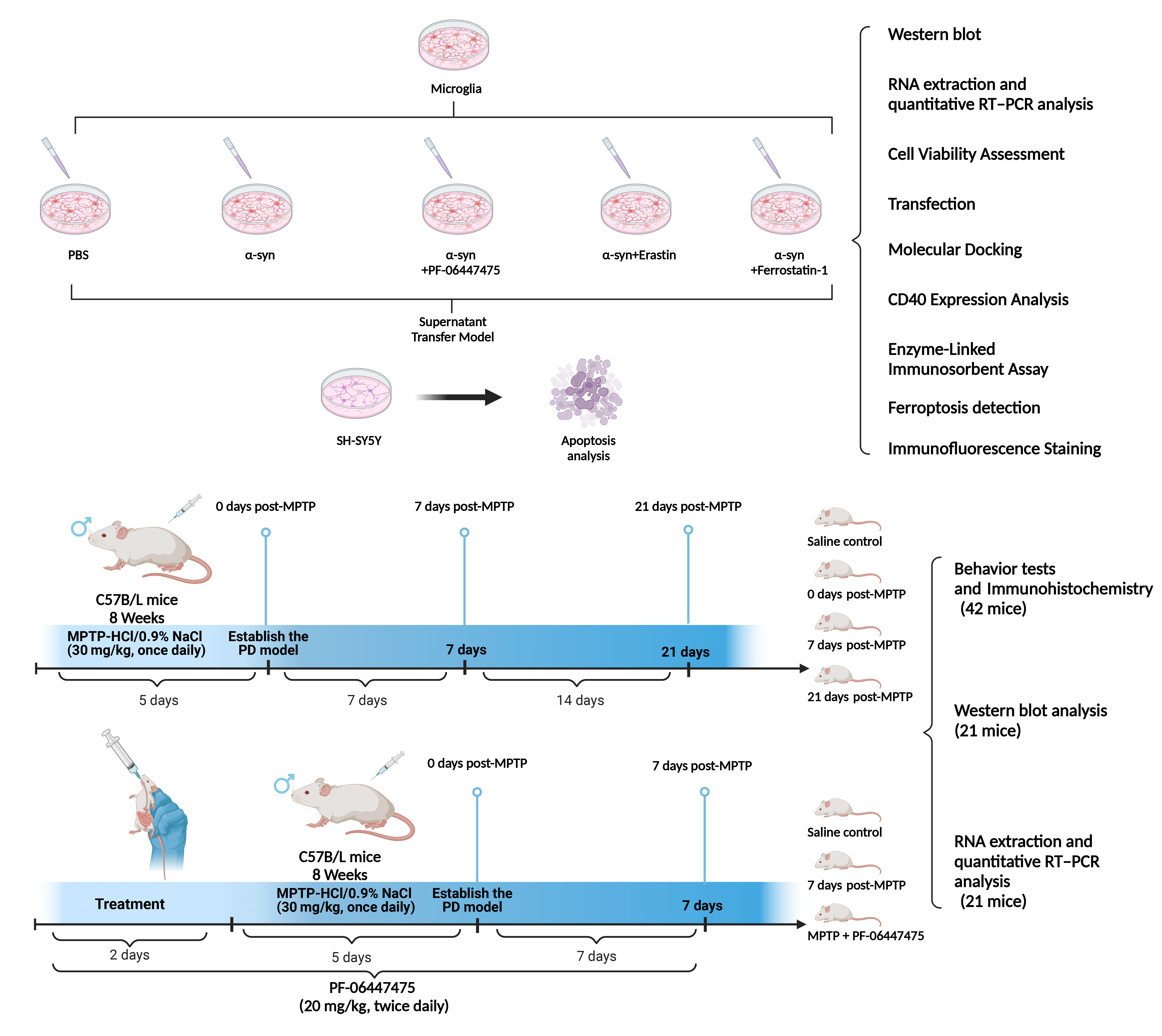

Supplement: Supplementary file 1 [file 10753_2025_2291_Fig12_ESM.png]

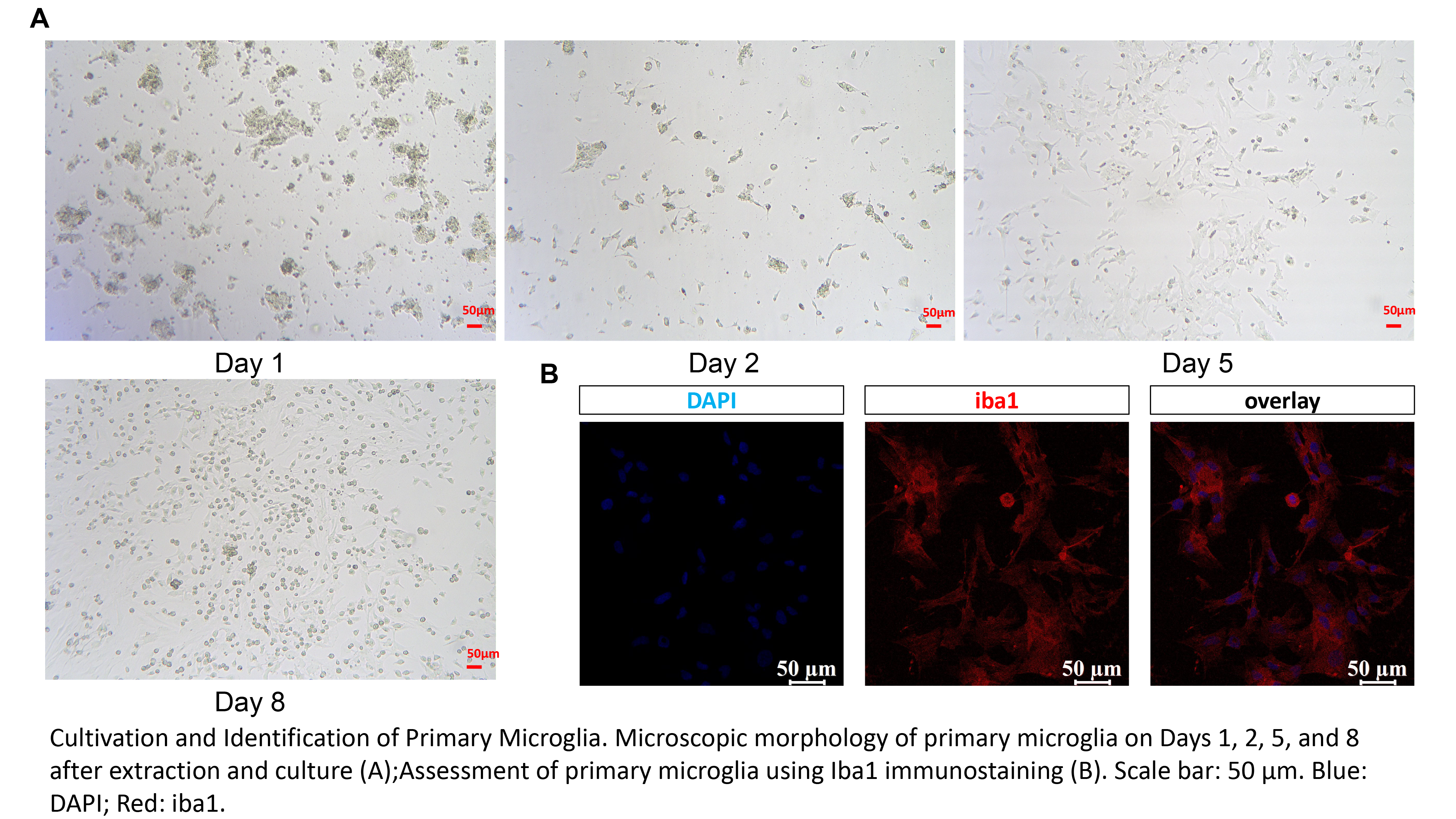

Supplement: Supplementary file 3 — Supplementary file 2 [file 10753_2025_2291_Fig13_ESM.png]

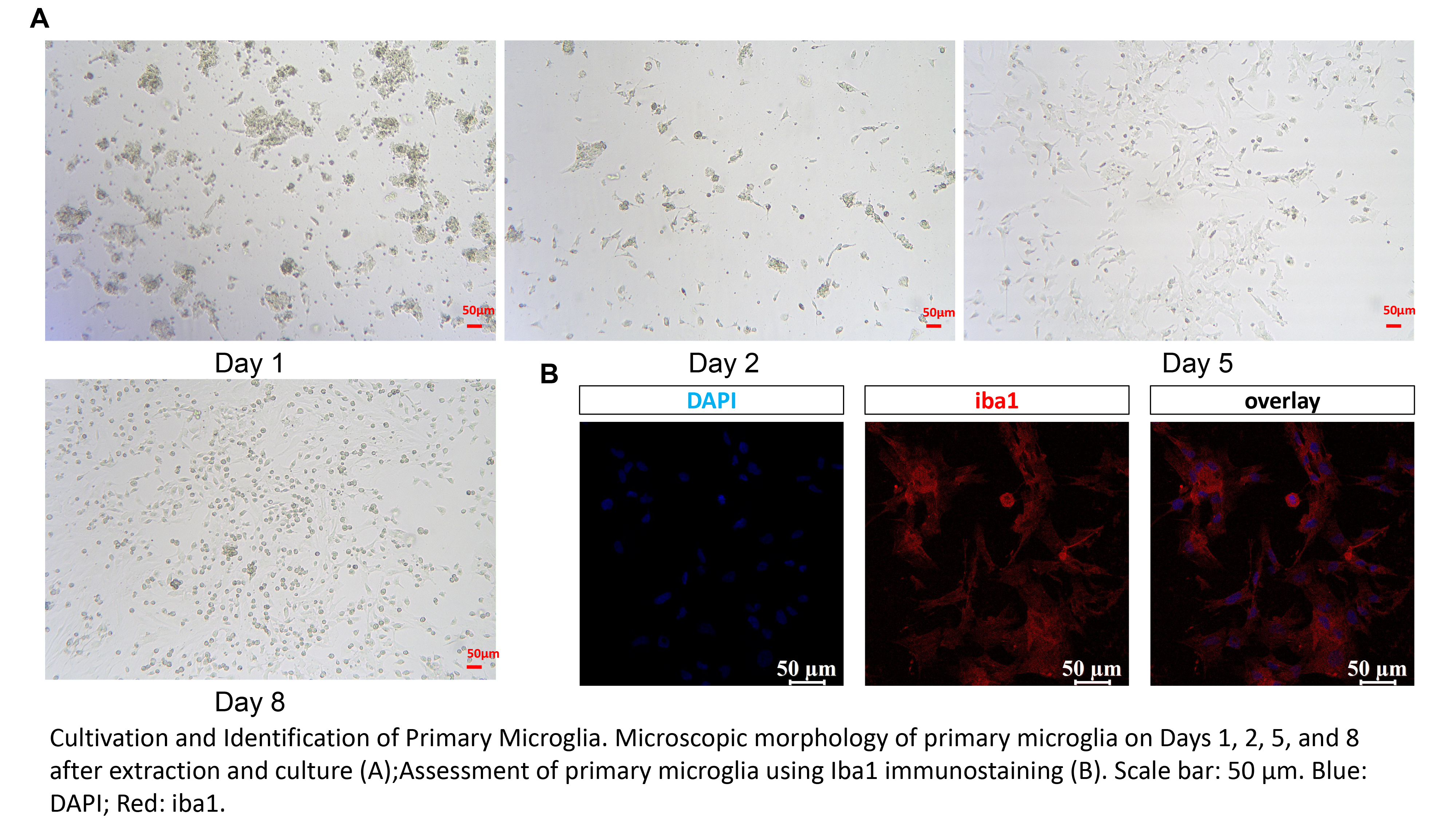

Supplement: Supplementary file 4 — High resolution image (41.1 MB) [file 10753_2025_2291_MOESM2_ESM.tif]

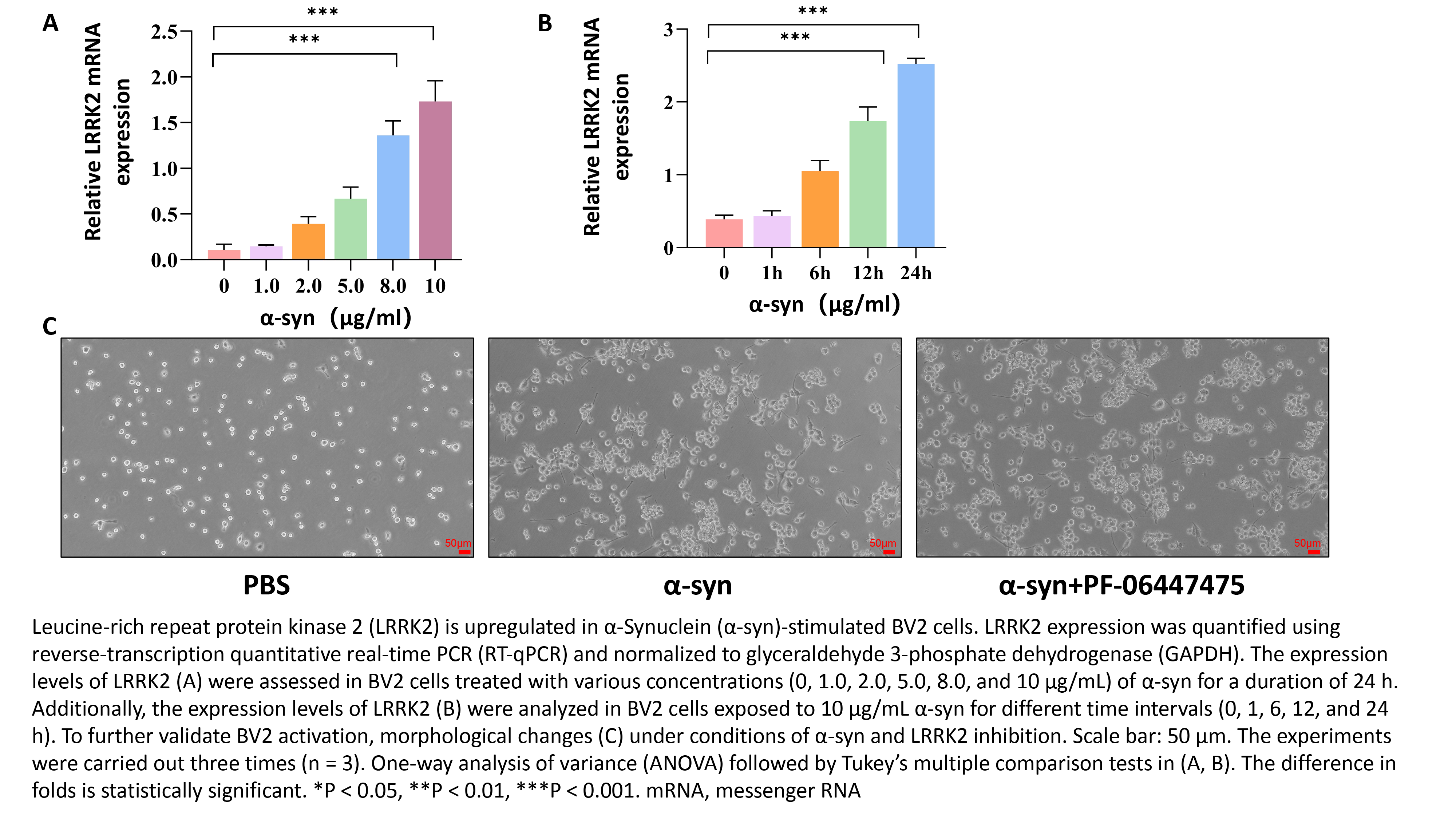

Supplement: Supplementary file 5 — Supplementary file 3 [file 10753_2025_2291_Fig14_ESM.png]

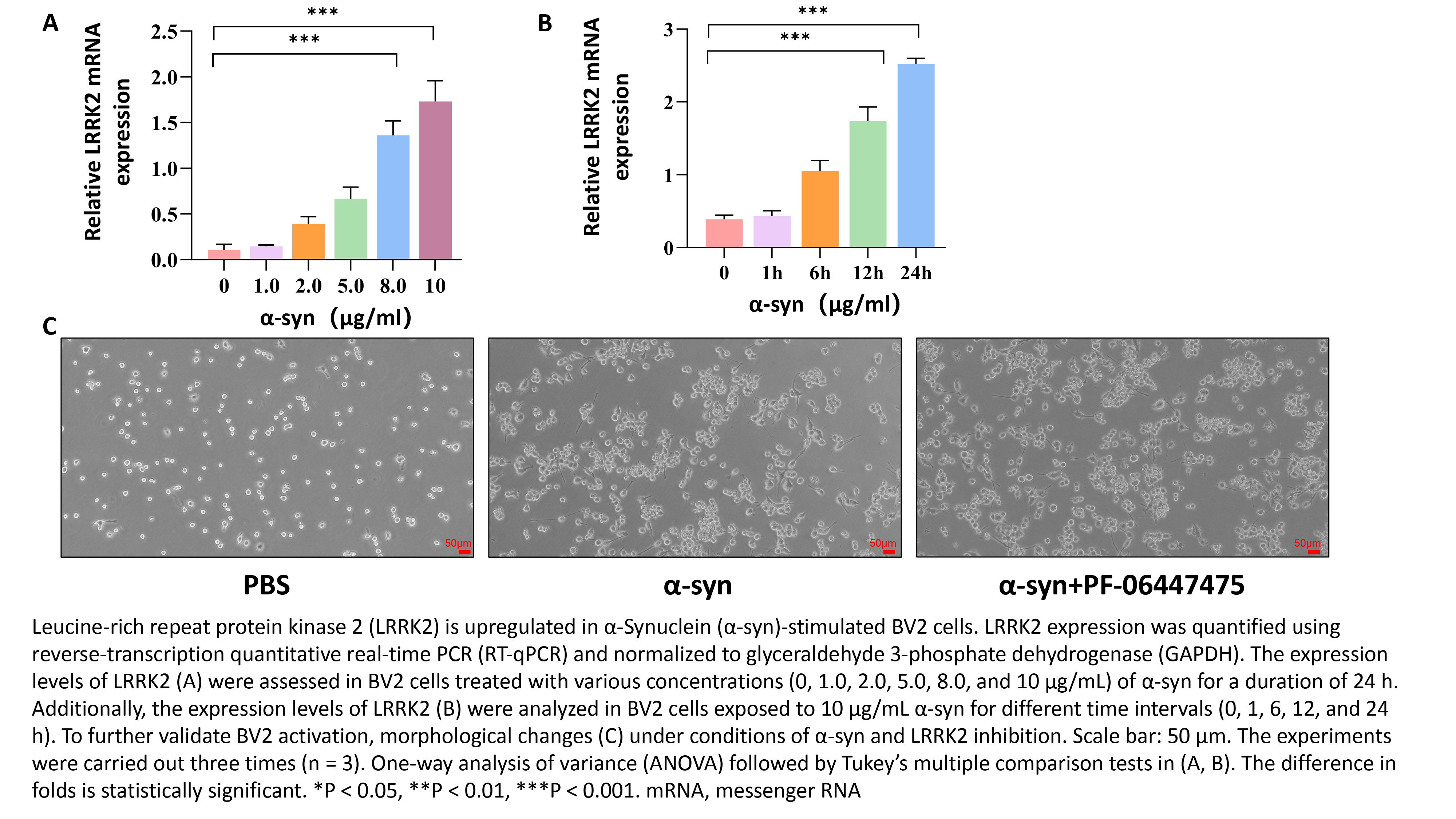

Supplement: Supplementary file 6 — High resolution image (33.3 MB) [file 10753_2025_2291_MOESM3_ESM.tif]
