# Supplementary material for: LRRK2 Mediates α-Synuclein-Induced Neuroinflammation and Ferroptosis through the p62-Keap1-Nrf2 Pathway in Parkinson’s Disease
Source: Inflammation. 2025 Apr 2;48(5):3666–91. doi: 10.1007/s10753-025-02291-8 (PMC12596413; doi:10.1007/s10753-025-02291-8)
Supplement: Supplementary file 7 — (DOCX 13.4 KB) [file 10753_2025_2291_MOESM4_ESM.docx]

**Supplementary Tables**

**Suppl. Table S1. Experimental groups and survival of mice.**

|  |  | **Behavior tests and Immunohistochemistry** | | | **Western blot analysis** | | | **RNA extraction and quantitative RT–PCR analysis** | | |
| --- | --- | --- | --- | --- | --- | --- | --- | --- | --- | --- |
|  | **Groups** | **Number** | **Dead** | **Survival** | **Number** | **Dead** | **Survival** | **Number** | **Dead** | **Survival** |
| **Experiment 1** | **Saline** | 7 | 1 | 6 | 3 | 0 | 3 | 4 | 1 | 3 |
|  | **0D after injected with MPTP-HCl** | 7 | 1 | 6 | 4 | 1 | 3 | 3 | 0 | 3 |
|  | **7D after injected with MPTP-HCl** | 8 | 2 | 6 | 4 | 1 | 3 | 5 | 2 | 3 |
|  | **21D after injected with MPTP-HCl** | 7 | 1 | 6 | 3 | 0 | 3 | 4 | 1 | 3 |
| **Experiment 2** | **Saline** | 6 | 0 | 6 | 4 | 1 | 3 | 3 | 0 | 3 |
|  | **7D after injected with MPTP-HCl** | 8 | 2 | 6 | 4 | 1 | 3 | 4 | 1 | 3 |
|  | **7D after injected with MPTP-HCl+ PF-06447475** | 7 | 1 | 6 | 3 | 0 | 3 | 4 | 1 | 3 |
